# Established and emerging pharmacologic options and unmet needs in HFpEF and HFmrEF

**DOI:** 10.1093/eschf/xvag056

**Published:** 2026-02-18

**Authors:** Andrew J Sauer, Jozine M ter Maaten, Gianluigi Savarese

**Affiliations:** Saint Luke's Mid America Heart Institute, 4401 Wornall Road, Kansas City, Missouri, MO 64111, USA School of Medicine, University of Missouri, Health Sciences Campus, 2411 Holmes Street, Kansas City, MO 64108, USA; University of Missouri, Kansas City, MO, USA; Department of Cardiology, University Medical Center Groningen, University of Groningen, Groningen, The Netherlands; Department of Clinical Science and Education, Södersjukhuset, Karolinska Institutet, Stockholm, Sweden

**Keywords:** GLP-1 RA, HFmrEF, HFpEF, MRA, RASi, SGLT2 inhibitor

## Abstract

A decline in mortality due to heart failure (HF) with reduced ejection fraction (HFrEF) has been attributed to effective guideline-directed medical therapies. But few effective therapies are available for HF with preserved ejection fraction (HFpEF), despite a high burden of HF events, or for HF with mildly reduced ejection fraction (HFmrEF). Novel therapies are needed for these HF subtypes.

Clinical trials have demonstrated the efficacy of sodium–glucose cotransporter 2 inhibitors for improving outcomes in HFpEF and HFmrEF. While renin–angiotensin system inhibitors, angiotensin receptor/neprilysin inhibitors, and steroidal mineralocorticoid receptor antagonists for HFpEF or HFmrEF have not demonstrated effects on primary trial outcomes, sub-analyses from large HF trials suggest they may reduce the risk of hospitalization for HF or mortality. Beta blockers may be beneficial for HFmrEF. Finerenone, a non-steroidal mineralocorticoid receptor antagonist, reduced HF events and cardiovascular deaths in participants with HF and ejection fraction ≥40% in the FINEARTS-HF trial, and is under evaluation for HFpEF and HFmrEF in the REDEFINE-HF and CONFIRMATION-HF trials.

As treatment for HFpEF and HFmrEF may be impacted by comorbidities, novel treatments could be tailored to specific phenotypes such as obesity and chronic kidney disease. Trials of glucagon-like peptide-1 receptor agonist (GLP-1 RA), semaglutide, and dual glucose-dependent insulinotropic polypeptide receptor agonist/GLP-1 RA, tirzepatide, for HFpEF with obesity, observed an impact on HF hospitalization events and quality of life.

Trials of selective mineralocorticoid modulator, balcinrenone, and aldosterone synthase inhibitor, vicadrostat, will address key evidence gaps and help improve outcomes for patients with HFpEF and HFmrEF.

## Graphical Abstract

**Graphical Abstract:**
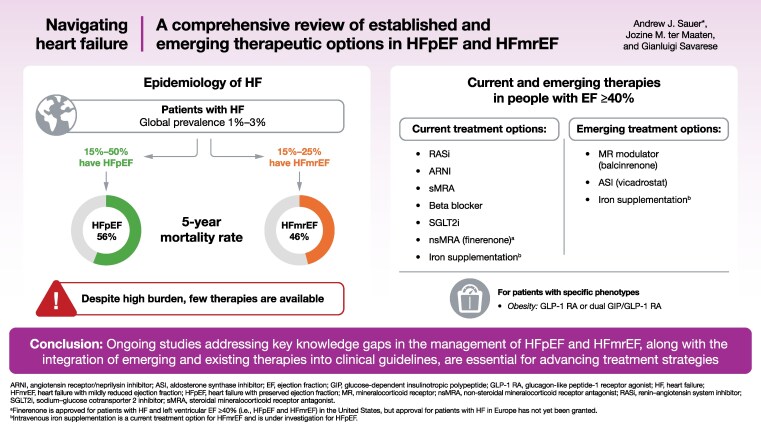

## Introduction

**Figure 1:**
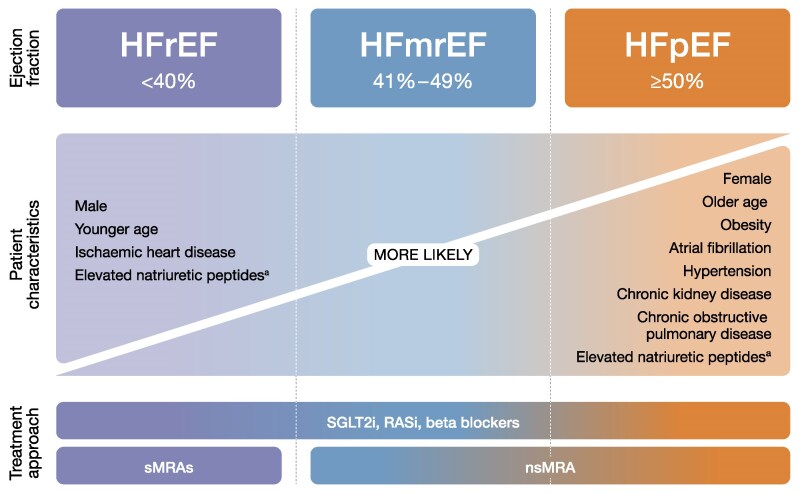
Classification, characteristics, and treatment outcomes of HF by phenotype.^2–4,6,7^ HF, heart failure; HFmrEF, heart failure with mildly reduced ejection fraction; HFpEF, heart failure with preserved ejection fraction; HFrEF, heart failure with reduced ejection fraction; nsMRA, non-steroidal mineralocorticoid receptor agonist; RASi, renin–angiotensin system inhibitor; SGLT2i, sodium–glucose cotransporter 2 inhibitor; sMRA, steroidal mineralocorticoid receptor antagonist. ^a^Elevated natriuretic peptides observed in HFrEF and HFpEF

Heart failure (HF) is a complex clinical disease characterized by structural or functional impairment of ventricular filling or ejection of blood.^1^ Guidelines generally classify HF according to left ventricular ejection fraction (EF) into three subsets (phenotypes)^2–5^ that differ in terms of their characteristics and outcomes (*Figure 1*).^6–8^

HF is a significant global health concern that affects more than 64 million people worldwide, with a prevalence estimated at 1%–3% in the general population.^9,10^ In registry studies of hospitalized patients, approximately 15%–50% of the HF population have HF with preserved EF (HFpEF) and approximately 15%–25% have HF with mildly reduced EF (HFmrEF).^9^ However, the prevalence of HFpEF and HFmrEF may be much higher than that of HF with reduced EF (HFrEF) in the broader population. In addition, HFpEF and HFmrEF may be underdiagnosed because they have minimally reduced or preserved EF and milder symptoms than HFrEF.^7,11^ While the prevalence of HFrEF is stable and the incidence is falling because of better treatments for ischaemic heart disease, the prevalence of HFpEF is rising as a consequence of an ageing population, the increasing burden of comorbidities, and improved disease awareness, and may soon exceed that of HFrEF.^9,11–13^

HF is associated with high morbidity and mortality, poor quality of life, and a substantial burden to the healthcare system.^9^ In the SwedeHF registry of people with HF enrolled at hospital discharge or in an outpatient setting, the 5-year mortality rate was higher in participants with HFpEF (56%) compared with those with HFrEF (45%) or HFmrEF (46%).^14^ Cardiovascular (CV) causes accounted for most deaths (HFrEF, 65%; HFpEF, 59%; HFmrEF, 58%), with ischaemic heart disease being the leading cause of CV death in all HF phenotypes. Non-CV deaths accounted for a higher proportion of deaths in HFpEF (39%) and HFmrEF (41%) compared with HFrEF (33%; *P* < .001 for both comparisons), and cancer was the most common cause of non-CV death in all HF phenotypes.^14^

All-cause hospitalization rates and healthcare costs for HF are substantial and are generally higher for people with HFrEF than HFpEF.^15,16^ However, total healthcare costs associated with people with HFpEF may be higher than those with HFrEF due to the presence of comorbidities.^17^

HFrEF is well characterized, and since most prior HF trials have focused on this population, guideline-recommended therapies are now available.^2,4^ For people with HFpEF and HFmrEF, who face an equally high burden of HF events, there are limited available therapies and a substantial unmet need remains.^2–4,18^

The objective of this review is to evaluate the evidence base for the current pharmacologic treatment options for people with HFpEF and HFmrEF, discuss the unmet therapeutic needs in this population, and review emerging treatments and therapeutic approaches.

## Evidence for current treatment options in people with HFpEF and HFmrEF (EF ≥40%)

Global guideline recommendations, as well as those for Europe, the USA, and Japan, describing current treatment options for people with HFpEF or HFmrEF are shown in *Table 1*, classified from I (strongest recommendation) to III (not recommended) based on the anticipated magnitude of treatment effect and benefit-to-risk ratio, with level of supporting evidence organized from A (highest) to C (lowest) based on scientific quality.^2–5,19^

**Table 1:** Summary of guideline recommendations for the treatment of HFpEF and HFmrEF^2–5,19^

Therapy	Organization	Guidance	Class^a,b^	Level^a,c^
HFpEF				
RASi	ESC	No recommendation	—	—
	AHA/ACC/HFSA	In selected people, an ARB may be considered to decrease hospitalizations, particularly among those with LVEF on the lower end of the spectrum	IIb	B-R
	JCS/JHFS	ARBs may be considered as an alternative to ARNI in symptomatic people with LVEF below normal to reduce the risk of HF hospitalizations	IIb	B-NR
	iCARDIO Alliance	Suggested to reduce the risk of HF hospitalization especially in people at the lower end of the LVEF spectrum	Suggested	—
ARNI	ESC	No recommendation	—	—
	AHA/ACC/HFSA	In selected people, it may be considered to decrease hospitalizations, particularly among those with LVEF on the lower end of the spectrum	IIb	B-R
	JCS/JHFS	May be considered in symptomatic people with LVEF below normal to reduce HF hospitalizations	IIa	B-NR
	iCARDIO Alliance	Recommended especially for people at the lower end of the LVEF spectrum. Particularly recommended in people with LVEF <58% and in women	Recommended	—
Beta blocker	ESC	No recommendation	—	—
	AHA/ACC/HFSA	No recommendation	—	—
	JCS/JHFS	No recommendation	—	—
	iCARDIO Alliance	No recommendation	—	—
sMRA	ESC	No recommendation	—	—
	AHA/ACC/HFSA	In selected people, it may be considered to decrease hospitalizations, particularly among those with LVEF on the lower end of the spectrum	IIb	B-R
	JCS/JHFS	Spironolactone or eplerenone may be considered in symptomatic people to reduce HF hospitalizations	IIb	B-NR
	iCARDIO Alliance	Spironolactone suggested to decrease HF hospitalization	Suggested	—
nsMRA	ESC	No recommendation	—	—
	AHA/ACC/HFSA	No recommendation	—	—
	JCS/JHFS	Finerenone may be considered in symptomatic people to reduce the risk of CV death or HF exacerbation events	IIa	B-R
	iCARDIO Alliance	Finerenone recommended to decrease HF hospitalization	Recommended	
SGLT2i	ESC	Dapagliflozin or empagliflozin recommended to reduce the risk of HF hospitalization or CV death	I	A
	AHA/ACC/HFSA	Can be beneficial for decreasing HF hospitalizations and CV mortality	IIa	B-R
	JCS/JHFS	Empagliflozin or dapagliflozin should be administered in symptomatic people to reduce the risk of HF hospitalization or CV death	I	A
	iCARDIO Alliance	Recommended to reduce the risk of HF hospitalization and CV death	Strongly recommended	—
GLP-1 RA	ESC	No recommendation	—	—
	AHA/ACC/HFSA	No recommendation	—	—
	JCS/JHFS	No recommendation	—	—
	iCARDIO Alliance	Tirzepatide or semaglutide recommended in people with obesity and HFpEF for weight loss and to improve symptoms and QoL	Strongly recommended	—
		Tirzepatide or semaglutide suggested in people with obesity and HFpEF to reduce the risk of HF hospitalization	Suggested	—
IV iron supplementation	ESC	No recommendation	—	—
	AHA/ACC/HFSA	No recommendation	—	—
	JCS/JHFS	No recommendation	—	—
	iCARDIO Alliance	No recommendation	—	—
HFmrEF^d^				
RASi	ESC	May be considered to reduce the risk of HF hospitalization and death	IIb	C
	AHA/ACC/HFSA	ARB may be considered to reduce the risk of HF hospitalization and CV mortality, particularly among people with LVEF on the lower end of the spectrum	IIb	B-NR
	JCS/JHFS	ARB may be considered in symptomatic people to reduce CV death or HF hospitalizations	IIb	B-NR
		ACEi may be considered in symptomatic people to reduce the risk of CV death or HF hospitalizations	IIb	C-LD
	iCARDIO Alliance	Recommended for ambulatory people to reduce mortality and morbidity, if ARNI is contraindicated	Strongly recommended	—
ARNI	ESC	May be considered to reduce the risk of HF hospitalization and death	IIb	C
	AHA/ACC/HFSA	May be considered to reduce the risk of HF hospitalization and CV mortality, particularly among people with LVEF on the lower end of the spectrum	IIb	B-NR
	JCS/JHFS	May be considered in symptomatic people to reduce the risk of CV death or HF hospitalizations	IIa	B-NR
	iCARDIO Alliance	Recommended for ambulatory people to reduce mortality and morbidity	Strongly recommended	—
		Recommended as a replacement therapy to reduce mortality and morbidity in people with NYHA II and III class symptoms who can tolerate RASi	Strongly recommended	—
Beta blocker	ESC	May be considered to reduce the risk of HF hospitalization and death	IIb	C
	AHA/ACC/HFSA	Evidence-based beta blockers for HFrEF may be considered to reduce the risk of HF hospitalization and CV mortality, particularly among people with LVEF on the lower end of the spectrum	IIb	B-NR
	JCS/JHFS	May be considered in symptomatic people to reduce the risk of CV death or HF hospitalizations	IIb	B-NR
	iCARDIO Alliance	Recommended (bisoprolol, carvedilol, nebivolol, or sustained-release metoprolol succinate) to reduce the risk of CV mortality and HF hospitalization	Strongly recommended	—
sMRA	ESC	May be considered to reduce the risk of HF hospitalization and death	IIb	C
	AHA/ACC/HFSA	May be considered to reduce the risk of HF hospitalization and CV mortality, particularly among people with LVEF on the lower end of the spectrum	IIb	B-NR
	JCS/JHFS	May be considered in symptomatic people to reduce risk of CV death or HF hospitalizations	IIb	B-NR
	iCARDIO Alliance	Recommended if eGFR >30 ml/min/1.73 m^2^ and potassium <5.0 mEq/L, to reduce morbidity and mortality	Strongly recommended	—
nsMRA	ESC	No recommendation	—	—
	AHA/ACC/HFSA	No recommendation	—	—
	JCS/JHFS	May be considered in symptomatic people to reduce the risk of CV death or HF exacerbation events	IIa	B-NR
	iCARDIO Alliance	Recommended if eGFR >30 ml/min/1.73 m^2^ and potassium <5.0 mEq/L, to reduce morbidity and mortality	Strongly recommended	—
SGLT2i	ESC	Dapagliflozin or empagliflozin recommended to reduce the risk of HF hospitalization or CV death	I	A
	AHA/ACC/HFSA	Can be beneficial for decreasing HF hospitalizations and CV mortality	IIa	B-R
	JCS/JHFS	Should be administered in symptomatic people to reduce the risk of HF hospitalization or CV death	I	A
	iCARDIO Alliance	Recommended to reduce the risk of HF hospitalization and CV death	Strongly recommended	
GLP-1 RA	ESC	No recommendation	—	—
	AHA/ACC/HFSA	No recommendation	—	—
	JCS/JHFS	No recommendation	—	—
	iCARDIO Alliance	No recommendation	—	—
IV iron supplementation	ESC	Ferric carboxymaltose or ferric derisomaltose recommended in symptomatic people to reduce the risk of HF hospitalization	IIa	A
	AHA/ACC/HFSA	No recommendation	—	—
	JCS/JHFS	No recommendation	—	—
	iCARDIO Alliance	IV ferric carboxymaltose or ferric derisomaltose recommended in iron deficiency to improve symptoms	Strongly recommended	—
		IV ferric carboxymaltose or ferric derisomaltose recommended in iron deficiency to reduce HF hospitalization and CV death	Recommended	—

A, data derived from multiple randomized clinical trials or meta-analyses; ACC, American College of Cardiology; ACEi, angiotensin-converting enzyme inhibitors; AHA, American Heart Association; ARB, angiotensin-receptor blocker; ARNI, angiotensin receptor/neprilysin inhibitor; B, data derived from a single randomized clinical trial or large non-randomized studies; C, consensus of opinion of the experts and/or small studies, retrospective studies, registries; CV, cardiovascular; EF, ejection fraction; eGFR, estimated glomerular filtration rate; ESC, European Society of Cardiology; GLP-1 RA, glucagon-like peptide-1 receptor agonist; HF, heart failure; HFmrEF, heart failure with mildly reduced ejection fraction; HFpEF, heart failure with preserved ejection fraction; HFrEF, heart failure with reduced ejection fraction; HFSA, Heart Failure Society of America; IV, intravenous; JCS, Japanese Circulation Society; JHFS, Japanese Heart Failure Society; LVEF, left ventricular ejection fraction; NR, non-randomized; nsMRA, non-steroidal mineralocorticoid receptor antagonist; NYHA, New York Heart Association; QoL, quality of life; R, randomized; RASi, renin–angiotensin system inhibitor; RCT, randomized controlled trial; SGLT2i, sodium-glucose cotransporter 2 inhibitor; sMRA, steroidal mineralocorticoid receptor antagonist.

^a^ESC, AHA/ACC/HFSA, and JCS/JHFS guidelines classify their recommendations from I to III based on the anticipated magnitude of treatment effect and benefit-to-risk ratio, with the level of supporting evidence organized from A to C based on scientific quality;

^b^Class I represents a strong recommendation; Class IIa, a moderate recommendation; Class IIb, a weak recommendation; and Class III, no recommendation with (in some cases) the potential to cause harm;

^c^Level A represents high-quality evidence (typically from high-quality RCTs or meta-analyses of these); level B, moderate evidence (typically from moderate-quality RCTs or meta-analyses of these, or from high-quality non-RCTs); and level C, limited evidence (typically from RCTs or non-RCTs with methodological limitations) or a consensus of expert opinion based on clinical experience;

^d^iCARDIO Alliance guidelines distinguishes therapeutically between HFrEF and HFpEF, and classifies HF with EF 40%–49% (defined elsewhere as HFmrEF) as HFrEF. These guidelines state that the absolute benefit of guideline-directed medical therapy may be somewhat less than in people with HF and EF <40%.^19^

Evidence from clinical trials supporting the effectiveness of current treatment options in people with HFpEF and HFmrEF is presented in *Table 2*.^20–45^ It should be noted that heterogeneity of trial populations regarding inclusion criteria (e.g. EF, age, hospitalization, and diuretic therapy), baseline characteristics, comorbidities, and background therapies limits comparisons between trials.

**Table 2:** Evidence for current treatment options in people with HFpEF and HFmrEF

Trial	Drug	Trial population and key inclusion criteria	Patient profile at baseline (mean, unless stated otherwise)	Key results (vs placebo unless stated otherwise)
RASi				
PEP-CHF^20^	Perindopril	Diastolic HF (*N* = 850) Aged ≥70 years Receiving diuretics for HF CV hospitalization within ≤6 months	Age (median): 75.0 years BMI: NR Diabetes: 20.6% EF (median): 64.5% CKD: NR	Primary outcome (composite of death or hospitalization) at 1 year: HR: 0.69 (95% CI 0.47–1.01); *P* = .055 Hospitalization for HF at 1 year: HR: 0.63 (95% CI 0.41–0.97); *P* = .033
CHARM-Preserved^21^	Candesartan	HFpEF and HFmrEF (*N* = 3023) EF >40% Aged ≥18 years History of CV hospitalizations	Age: 67.2 years BMI: 29.2 kg/m^2^ Diabetes: 28.4% EF 41%–49%: 35.4% CKD: NR	Primary outcome (composite of CV death or hospitalization for HF) during median follow-up of 36.6 months: HR: 0.89 (95% CI 0.77–1.03); *P* = .118
I-PRESERVE^22^	Irbesartan	HFpEF (*N* = 4128) EF ≥45% Aged ≥60 years HF hospitalization within ≤6 months	Age: 72.0 years BMI: 29.7 kg/m^2^ Diabetes: 27.5% EF ≥45% CKD: NR	Primary outcome (composite of all-cause death or hospitalization for CV disease) during mean follow-up of 49.5 months: HR: 0.95 (95% CI 0.86–1.05); *P* = .35 Hospitalization for HF: HR: 0.95 (95% CI 0.85–1.08); *P* = .50 HFpEF subgroup with obesity, hyperlipidaemia, diabetes, anaemia, renal insufficiency, and low NT-proBNP^23^ HR: 0.45 (95% CI 0.21–0.95), *P* = .037
DIG-Ancillary^24^	Digoxin	HFpEF (*N* = 988) EF >45% Normal sinus rhythm	Age: 66.8 years BMI: NR Diabetes: 28.8% EF 45%–49%: 27.2% EF ≥50%: 72.8% CKD: NR	Primary outcome (composite of HF mortality or hospitalization for HF) during mean follow-up of 37 months: HR: 0.82 (95% CI 0.63–1.07); *P* = .136 Hospitalization for worsening HF: HR: 0.79 (95% CI 0.59–1.04); *P* = .094
CHARM Programme^25^	Candesartan	HFmrEF (*N* = 1322) EF 40%–49%	Age: 65 years BMI: 27.8 kg/m^2^ Diabetes: 28.6% EF 40%–49% CKD: NR	Primary outcome (composite of CV death or hospitalization for HF) during mean follow-up of 2.9 years: HR: 0.76 (95% CI 0.61–0.96); *P* = .02
ARNI				
PARAGON-HF^26^	Sacubitril/ valsartan vs valsartan	HFpEF (*N* = 4796) EF ≥45% Aged ≥50 years Receiving diuretics in the month before enrolment Elevated NT-proBNP^a^	Age: 72.7 years BMI: 30.2 kg/m^2^ Diabetes: 43.0% EF 45%–57%: 52.0% EF >57%: 48.0% CKD: NR	Primary outcome (composite of CV death or hospitalization for HF) during median follow up of 35 months: RR: 0.87 (95% CI 0.75–1.01); *P* = .06 Primary outcome by subgroup EF ≤57%: RR: 0.78 (95% CI 0.64–0.95), Primary outcome by subgroup EF >57%: RR: 1.00 (95% CI 0.81–1.23); *P* = NR Hospitalization for HF: RR: 0.85 (95% CI 0.72–1.00)
PARAGON-HF and PARADIGM-HF pooled analysis^26,27^	Sacubitril/ valsartan vs valsartan or enalapril	HFpEF, HFmrEF, and HFrEF (*N* = 13 195) PARAGON-HF: EF ≥45% Aged ≥50 years Receiving diuretics in the month before enrolment Elevated NT-proBNP^a^ PARADIGM-HF: EF ≤40% Aged ≥18 years Receiving diuretics in the month before enrolment Elevated NT-proBNP or BNP^b^	Age: 68.0 years BMI: 29.1 kg/m^2^ Diabetes: 37.7% EF 32.5%–42.5%: 23.8% EF >42.5%–52.5%: 10.8% EF >52.5%: 25.5% CKD: NR	Total hospitalization for HF and CV death by EF during median follow-up of 27 months and 35 months: >32.5%–42.5%: HR: 0.82 (95% CI 0.68–0.99); *P* = .041 > 52.5%–62.5%: HR: 0.83 (95% CI 0.66–1.04); *P* = .11 > 62.5%: HR: 1.06 (95% CI 0.79–1.41); *P* = .72
PARAGLIDE-HF^28^	Sacubitril/ valsartan vs valsartan	HFpEF and HFpEF (*N* = 466) EF >40% Elevated NT-proBNP or BNP^c^ Aged ≥18 years Current HF hospitalization or recent, within 30 days of discharge for HF event requiring intravenous diuretic treatment	Age: 71.5 years BMI: 33.0 kg/m^2^ Diabetes: 48.5% EF 41%–49%: 23.0% EF ≥50%: 77.0% CKD: NR	Hierarchical composite endpoint (CV death, hospitalization for HF, urgent HF visits, change in NT-proBNP) during median follow-up of 5.9 months: win ratio: 1.19 (95% CI 0.93–1.52); *P* = .16 EF ≤60%: win ratio: 1.46 (95% CI 1.09–1.95)
sMRA				
TOPCAT^29^	Spironolactone	HFpEF (*N* = 3445) EF ≥45% Aged ≥50 years Hospitalization ≤12 months, or NT-proBNP ≥360 pg/ml or BNP ≥100 pg/ml ≤60 days before randomization	Age (median): 68.7 years BMI (median): 31.0 kg/m^2^ Diabetes: 32.5% EF 45%–49%: 15.1% EF ≥50%: 84.9% CKD (eGFR <60 ml/min/1.73 m^2^): 38.7%	Primary outcome (death from CV causes, aborted cardiac arrest, or hospitalization for HF) during mean follow up of 3.3 years: HR: 0.89 (95% CI 0.77–1.04); *P* = .14 EF subgroup analysis^30^: EF <50%: HR: 0.72 (95% CI 0.50–1.05), EF 50–54.99%: HR: 0.85 (95% CI 0.61–1.18), EF 55–59.99%: HR: 0.94 (95% CI 0.68–1.29), EF ≥60%: HR: 0.97 (95% CI 0.76–1.23), interaction *P* = .046 Post-hoc analysis by region^31^: Russia/Georgia: HR: 1.10 (95% CI 0.79–1.51), Americas: HR: 0.82 (95% CI 0.69–0.98), interaction *P* = .12 Hospitalization for HF: HR: 0.83 (95% CI 0.69–0.99); *P* = .04 EF subgroup analysis^30^: EF <50%: HR: 0.76 (95% CI 0.46–1.27), EF 50–54.99%: HR: 0.70 (95% CI 0.47–1.04), EF 55–59.99%: HR: 0.73 (95% CI 0.49–1.08), EF ≥60%: HR: 0.98 (95% CI 0.74–1.30), interaction *P* = .039 HFpEF phenotype of people who were older, obese, diabetic, and with left ventricular hypertrophy^32^ HR: 0.75 (95% CI 0.59–0.95)
SPIRIT-HF^33^	Spironolactone	HFpEF and HFmrEF (*N* = 743) EF ≥40% Aged ≥50 years Elevated NT-proBNP^d^ HF hospitalization or intravenous diuretics within ≤12 months	NA—trial terminated	Primary outcome: Composite of CV death or hospitalization for HF Secondary outcomes: Rate of CV death Rate of HF hospitalization Rate of all-cause death Rate of all-cause hospitalization
SPIRRIT-HFpEF^34^	Spironolactone/eplerenone	HFpEF and HFmrEF (estimated *N* = 2400) EF ≥40% Aged ≥50 years Elevated NT-proBNP or BNP^e^	NA—trial ongoing	Primary outcome: Composite of CV death or hospitalization for HF Secondary outcomes: Time to CV death or HF hospitalization Time to all-cause death Time to all-cause hospitalization
Beta blocker				
Meta-analysis^35^	Beta blockers	HFpEF, HFmrEF, and HFrEF (*N* = 14 262)	Age (median): EF 40%–49%, 71 years EF ≥50%: 75 years BMI (median): EF ≥40%, 27 kg/m^2^ Diabetes: 23.1% EF 40%–49%: 4.0% EF ≥50%: 1.7% CKD: NR	All-cause mortality by EF for 1.3 years median follow-up: EF 40%–49%: HR: 0.59 (95% CI 0.34–1.03); *P* = .066 EF ≥50%: HR: 1.79 (95% CI 0.78–4.10); *P* = .17 CV death by EF for 1.3 years median follow-up: 40%–49%: HR: 0.48 (95% CI 0.24–0.97); *P* = .04 ≥ 50%: HR: 1.77 (95% CI 0.61–5.14); *P* = .29
SGLT2i				
EMPEROR-Preserved^36,37^	Empagliflozin	HFpEF and HFpEF (*N* = 5988) EF >40% Aged ≥18 years Elevated NT-proBNP^f^	Age: 71.9 years BMI: 29.8 kg/m^2^ Diabetes: 49.1% EF 40%–49%: 33.1% EF ≥50%: 66.9% CKD (eGFR <60 ml/min/1.73 m^2^): 49.9%	Primary outcome (composite of CV death or hospitalization for HF) for 26.2 months median follow-up: HR: 0.79 (95% CI 0.69–0.90); *P* < .001 EF <50%: HR: 0.71 (95% CI 0.57–0.88), EF ≥50%–<60%: HR: 0.80 (95% CI 0.64–0.99) EF ≥60%: HR 0.87 (95% CI 0.69–1.10) Hospitalization for HF: HR: 0.73 (95% CI 0.61–0.88); *P* < .001
DELIVER^38^	Dapagliflozin	HFpEF and HFpEF (*N* = 6263) EF >40% Aged ≥40 years Elevated NT-proBNP^g^	Age: 71.7 years BMI: 29.9 kg/m^2^ Diabetes (T2D): 44.8% EF 40%–49%: 33.8% EF ≥50%: 66.2% CKD (eGFR <60 ml/min/1.73 m^2^): 49.0%	Primary outcome (composite of CV death or worsening HF [hospitalization or urgent visit]) for 2.3 years median follow-up: HR: 0.82 (95% CI 0.73–0.92); *P* < .001 EF <50%: RR: 0.87 (95% CI 0.72–1.04), EF ≥50%–59%: RR: 0.79 (95% CI 0.65–0.97), EF ≥60%: RR: 0.78 (95% CI 0.62–0.98); interaction *P* = NR Worsening HF: HR: 0.79 (95% CI 0.69–0.91)
PRESERVED-HF^39^	Dapagliflozin	HFpEF (*N* = 324) EF ≥45% Aged ≥18 years Elevated NT-proBNP or BNP^h^ Requirement for diuretic therapy HF hospitalization or urgent HF visit with intravenous diuretic within ≤12 months	Age (median): 70.0 years BMI (median): 34.9 Diabetes (T2D): 55.9%	Improved KCCQ-CSS vs placebo at 12 weeks: 5.8 points (95% CI 2.3–9.2); *P* = .001 Improved 6MWD: mean effect size 20.1 m (95% CI 5.6–34.7, *P* = .007)
nsMRA				
FINEARTS-HF^40,41^	Finerenone	HFpEF and HFmrEF (*N* = 6001) EF ≥40% Aged ≥40 years Elevated NT-proBNP or BNP^a^ Receiving diuretics for ≥30 days prior to randomization	Age: 72.0 years BMI: 29.9 kg/m^2^ Diabetes (T2D): 40.6% EF <50%: 36.2% EF ≥50%: 63.7% CKD (eGFR <60 ml/min/1.73 m^2^): 48.1%	Primary outcome (composite of CV death or worsening HF [hospitalization or urgent visit]) for 32 months median follow-up: HR: 0.84 (95% CI 0.74–0.95); *P* = .007 EF <60%: RR: 0.82 (95% CI 0.71–0.94), EF ≥60%: RR: 0.94 (95% CI 0.70–1.26); interaction *P* = NR Worsening HF events: rate ratio: 0.82 (95% CI 0.71–0.94); *P* = .006 CV death: HR: 0.93 (95% CI 0.78–1.11) Mean change in KCCQ-TSS at Month 12: 1.62 points (95% CI 0.69–2.56); *P* < .001^42^ Receiving SGLT2i at baseline: RR: 0.83 (95% CI 0.60–1.16); Not receiving SGLT2i at baseline: RR: 0.85 (95% CI 0.74–0.98); *P*_interaction_ = .76^43^
REDEFINE-HF^44^	Finerenone	HFpEF and HFpEF (estimated *N* = 5200) EF ≥40% Aged ≥18 years Elevated NT-proBNP or BNP^b^ Current or recent HF hospitalization	NA—trial ongoing	Primary outcome: Composite of total (first and subsequent) HF hospitalizations, urgent visits for worsening HF, and CV death Secondary outcomes: Time to first occurrence of the composite of CV death or HF event Total HF events, time to CV death, time to all-cause mortality Change from baseline in KCCQ-TSS at Month 6
CONFIRMATION-HF^45^	Finerenone plus empagliflozin	HF (estimated *N* = 1500) Aged ≥18 years Elevated NT-proBNP or BNP^b^ Current or recent HF hospitalization	NA—trial ongoing	Primary outcome: Hierarchical composite of time to death from any cause, number of HF events, time to first HF event, difference of 5 points or greater on the KCCQ-TSS Secondary outcomes: Time to first occurrence of all-cause mortality or HF event (hospitalization for HF or urgent visit due to HF) Total (first and recurrent) HF events Change from baseline in KCCQ-TSS at Month 6

6MWD, 6-minute walking distance; AF, atrial fibrillation; ARNI, angiotensin receptor/neprilysin inhibitor; BMI, body mass index; BNP, B-type natriuretic peptide; CI, confidence interval; CKD, chronic kidney disease; CV, cardiovascular; EF, ejection fraction; eGFR, estimated glomerular filtration rate; HF, heart failure; HFmrEF, heart failure with mildly reduced ejection fraction; HFpEF, heart failure with preserved ejection fraction; HFrEF, heart failure with reduced ejection fraction; HR, hazard ratio; KCCQ-CSS, Kansas City Cardiomyopathy Questionnaire Clinical Summary Score; KCCQ-TSS, Kansas City Cardiomyopathy Questionnaire Total Symptom Score; NR, not reported; NT-proBNP, N-terminal pro–B-type natriuretic peptide; RASi, renin–angiotensin system inhibitor; RR, risk ratio; SGLT2i, sodium–glucose cotransporter 2 inhibitor; sMRA, steroidal mineralocorticoid receptor antagonist; T2D, type 2 diabetes.

^a^NT-proBNP >300 pg/ml (>900 pg/ml if in AF) if not hospitalized for HF within 9 months or >200 pg/ml (>600 pg/ml if in AF) if hospitalized for HF within 9 months.

^b^NT-proBNP ≥600 pg/ml or BNP ≥150 pg/ml (≥400 pg/ml or ≥100 pg/ml, respectively, if hospitalized in the previous year).

^c^NT-proBNP ≥500 pg/ml or BNP ≥150 pg/ml (≥1000 pg/ml or ≥300 pg/ml, respectively, if in AF).

^d^NT-proBNP >300 pg/ml (>900 pg/ml if in AF) or BNP >80 pg/ml (>250 pg/ml if in AF) only if NT-proBNP not available.

^e^NT-proBNP >300 ng/L or BNP >100 pg/ml (>750 ng/L or >250 pg/ml, respectively, if in AF) at most recent blood sample (adjustments may be made for BMI) or NT-proBNP >1200 ng/L or BNP >400 pg/ml within the last 12 months even if most recent value was lower.

^f^NT-proBNP >300 pg/ml (>900 pg/ml if in AF).

^g^NT-proBNP >300 pg/ml (>600 pg/ml if in AF).

^h^NT-proBNP ≥225 pg/ml or BNP ≥75 pg/ml (≥375 pg/ml or ≥100 pg/ml, respectively, if in AF).

### Renin–angiotensin system inhibitors (RASis), angiotensin receptor/neprilysin inhibitors (ARNIs), and steroidal mineralocorticoid receptor (MR) antagonists (sMRAs)

RASis target the neurohormonal activation that occurs in HF in response to a fall in cardiac output.^46^ Renin–angiotensin system activation is a key feature of HFrEF,^47^ is related to hypertension, which is prevalent in HFpEF,^48^ and is an important contributory factor in the development of CV disease in people with chronic kidney disease (CKD).^49^ Despite this, none of the large randomized controlled trials conducted with RASis, ARNIs, or sMRAs in people with HFpEF, including PEP-CHF (perindopril), CHARM-Preserved (candesartan), I-PRESERVE (irbesartan), TOPCAT (spironolactone), DIG-Ancillary (digoxin), and PARAGON-HF (sacubitril/valsartan), achieved their primary endpoint (*Table 2*).^20–22,24,26,29^ Heterogeneity of the trial populations increases complexity in evaluating the reasons for the failed primary endpoints when comparing the trials. However, perindopril reduced hospitalization for HF compared with placebo in PEP-CHF.^20^ A similar trend was shown in the PARAGON-HF trial, although no statistically significant difference was seen (*Table 2*).^26^ Subgroup analyses of I-PRESERVE and TOPCAT suggest that people with HFpEF and comorbidities, such as obesity and diabetes, may derive greater benefit from RASis or sMRA therapy than those with HFpEF without these comorbidities (*Table 2*).^23,32^

There have been no dedicated trials for RASis, ARNIs, and sMRAs in HFmrEF; however, sub-analyses from the CHARM, PARAGON-HF, and TOPCAT trials have suggested some benefit in this population.^25,26,29^

In CHARM, candesartan reduced the primary outcome (CV death or HF hospitalization) in participants with HFmrEF (17% of the CHARM programme population) as compared with placebo (*Table 2*).^25^

In PARAGON-HF, sacubitril/valsartan did not significantly reduce hospitalizations for HF and CV death compared with valsartan overall; however, a greater benefit was observed in people with an EF of 45%–57% compared with those with an EF of >57% (*Table 2*).^26^ A pooled analysis of PARAGON-HF (EF eligibility, ≥45%) and PARADIGM-HF (EF eligibility, ≤40%) suggested some benefit of sacubitril/valsartan in people with HFmrEF in terms of first hospitalization for HF or CV death (HR: 0.81, 95% CI 0.64–1.03; *P* = .09).^27^ In the PARAGLIDE-HF study, although no significant clinical benefit was seen in the overall cohort of participants with EF >40%, when limiting the analysis to those with EF ≤60% a notable benefit was observed (*Table 2*).^28^

In TOPCAT, spironolactone did not significantly reduce the risk of the primary composite outcome of CV death, aborted cardiac arrest, or hospitalization for HF compared with placebo in people with an EF ≥45% (15% with EF ≥45 to <50%), over a mean follow-up of 3 years.^29^ However, people with an EF at the lower end of the spectrum (<50%) were more likely to benefit from spironolactone with respect to the primary composite outcome and hospitalization for HF (*Table 2*).^30^

Post-hoc analyses of data from TOPCAT demonstrated variations in baseline characteristics, response to spironolactone, and spironolactone metabolite concentrations between the two regions (Russia and Georgia compared with the Americas [the USA, Canada, Brazil, and Argentina]).^31,50^ Taken together, these findings called into question the study’s conduct at some sites, and suggested that a significant proportion of participants in Russia/Georgia randomized to spironolactone may not have had HF or taken randomized treatment.^50^ In contrast with the finding in the study population overall, the rate of the primary outcome was nominally significantly reduced by spironolactone compared with placebo in the Americas (HR: 0.82, 95% CI 0.69–0.98; *P* = .026).^31^

An individual patient-level meta-analysis of four randomized controlled trials, including TOPCAT, demonstrated that sMRAs reduced the risk of HF hospitalization or CV death compared with placebo in people with HF across the EF spectrum (HR: 0.77, 95% CI 0.72–0.83).^51^

The SPIRIT-HF and SPIRRIT-HFpEF studies were designed to assess whether the initiation of spironolactone or eplerenone improves outcomes in people with HFpEF and HFmrEF (*Table 2*). SPIRIT-HF, a double-blind, randomized, placebo-controlled interventional Phase 3 study, enrolled 743 participants but was terminated.^33^ SPIRRIT-HFpEF is a registry-randomized trial evaluating data from 2400 participants; both studies have a primary composite endpoint of hospitalization for HF or CV death.^34^ A positive finding in SPIRRIT-HFpEF would provide additional support for the use of sMRAs in people with HFpEF and HFmrEF.

Most sMRAs are associated with hyperkalaemia due to the role of aldosterone in controlling potassium homeostasis.^52^ In TOPCAT, spironolactone was associated with a doubling of the incidence of hyperkalaemia (potassium ≥5.5 mmol/L 18.7 vs 9.1% in the placebo group) in people with HFpEF or HFmrEF.^29^ The incidence of hypokalaemia (potassium <3.5 mmol/L), which is also associated with increased risk of mortality,^53^ was reduced by spironolactone compared with placebo (16.2 vs 22.9%, respectively).^29^ Serum potassium should be monitored within 1 week of treatment initiation and during treatment with these agents.^54,55^ Hyperkalaemia mitigation strategies might include combining an sMRA with an SGLT2i, use of potassium binders, and use of loop or thiazide diuretics.^56^

Based on the findings of the studies described above, global guidelines and those in the USA and Japan recommend that angiotensin receptor blockers, ARNIs, or sMRAs may be considered for specific groups of people with HFpEF, whereas there is no such recommendation in Europe (*Table 1*).^2–5,19^ For people with HFmrEF, guidelines state that treatment with RASis, ARNIs, or sMRAs may be considered to reduce the risk of hospitalization or death (*Table 1*).^2–5,19^

### Beta blockers

A meta-analysis of 11 studies demonstrated no significant improvement in all-cause and CV mortality with beta blockers compared with placebo in people with HF and EF ≥50% (*Table 2*).^35^ In addition, beta blockers did not improve prognosis in people with HFpEF and atrial fibrillation, a frequent comorbidity of HFpEF.^35^ Observational data from a large, real-world cohort of outpatients ≥65 years of age found that beta blocker use was associated with an increased risk of hospitalization in people with HFpEF, notably when EF exceeded 60%.^57^ The lack of supporting data means that guidelines do not include recommendations for the use of beta blockers in people with HFpEF (*Table 1*).^2,4,5,19^

The meta-analysis of studies of beta blockers in HF demonstrated a significant reduction in all-cause and CV mortality compared with placebo in those with an EF of 40%–49% (*Table 2*).^35^ As many people with HFmrEF may have another CV indication, HF guidelines state that use of a beta blocker may be considered to reduce the risk of hospitalization for HF or death in people with HFmrEF (*Table 1*).^2,4,19^

### Sodium–glucose cotransporter 2 inhibitors (SGLT2is)

Of the currently approved HF therapies, only SGLT2is have convincingly shown a benefit in people with EF >40%.^36,38,58^ In the EMPEROR-Preserved trial in people with HF and an EF >40% (33.1% with EF 40%–49%), empagliflozin was associated with a 21% reduction in the primary composite endpoint of hospitalization for HF or CV death compared with placebo (*P* < .001; *Table 2*), an effect that was consistent in people with HFpEF and HFmrEF.^36,59^ DELIVER assessed the efficacy and safety of dapagliflozin in people with HFpEF and HFmrEF (33.8% with EF 40% to ≤49%).^38^ Dapagliflozin was associated with an 18% reduction (*P* < .001) for the primary composite endpoint of worsening HF or CV death compared with placebo, this effect was consistent regardless of EF at baseline (*Table 2*).^38^

SGLT2is have also been reported to improve patient-reported outcomes. The PRESERVED-HF study showed that dapagliflozin improved the Kansas City Cardiomyopathy Questionnaire Clinical Summary Score (KCCQ-CSS; ≥5 points is clinically meaningful) and 6-minute walk distance compared with placebo in participants with HFpEF (*Table 2*).^39,60^

Analysis of data from eight studies that evaluated the effect of SGLT2is on adverse events (AEs) in people with HFmrEF or HFpEF showed that rates of volume depletion, kidney injury, drug discontinuation, ketoacidosis, hypoglycaemia, amputation, and urinary tract infections were similar compared with placebo.^58^ Furthermore, the risk of serious AEs was significantly lower in those receiving SGLT2is vs placebo (risk ratio: 0.94, 95% CI 0.91–0.98; *P* = .07).^58^

Supported by the findings of the EMPA-REG OUTCOME,^61^ EMPA-KIDNEY,^62^ DECLARE-TIMI 58,^63^ DAPA-CKD,^64^ CANVAS,^65^ and CREDENCE^66^ studies, SGLT2is are also recommended by the American Diabetes Association and the Kidney Disease: Improving Global Outcomes CKD Work Group for reducing CV risk and progression of CKD in people with type 2 diabetes (T2D) and CKD,^67–69^ conditions that frequently coexist with HFpEF and HFmrEF.^70–72^

### Non-steroidal MR antagonists (nsMRA)

Finerenone is an nsMRA with distinct physiochemical properties from sMRAs. Compared with spironolactone and eplerenone, it exhibits higher selectivity for the MR, a shorter half-life, lacks active metabolites, achieves equal distribution between the heart and kidneys, and utilizes distinct transcriptional coactivators.^73,74^ Finerenone acts as an inverse agonist ligand, reducing MR cofactor recruitment even in the absence of aldosterone.^73^ Additionally, finerenone may offer more potent anti-inflammatory and anti-fibrotic effects related to cardiac MR activation due to its more balanced distribution between the heart and kidneys.^73,74^ Finerenone's shorter plasma half-life compared to sMRAs may help lower the risk of hyperkalaemia.^73^ Finerenone is indicated to reduce CV risk and CKD progression in people with CKD associated with T2D^75^ following the results of the FIDELIO-DKD and FIGARO-DKD studies.^76,77^ It has recently been approved for patients with HF and left ventricular EF ≥40% (i.e. HFpEF and HFmrEF) in the USA;^75^ approval for patients with HF in Europe has not yet been granted.

In FINEARTS-HF, finerenone was associated with a 16% reduction (*P* = .007) in the primary composite endpoint of total (first and recurrent) HF events and CV death in participants with HFpEF and HFmrEF (36.2% with EF <50%); this effect was consistent in people with an EF of <60% and ≥60% (*Table 2*).^40^ Hyperkalaemia (potassium >5.5 mmol/L) occurred more frequently in the finerenone group (14.3 vs 6.9% with placebo). Similarly, investigator-reported cases of hyperkalaemia were higher with finerenone (9.7 vs 4.2% with placebo). Hospitalizations due to hyperkalaemia were rare in both groups (0.5% in the finerenone group vs 0.2% in the placebo group), with no events leading to death.^40^

A prespecified analysis of FINEARTS-HF demonstrated that the benefit of finerenone was similar in participants treated or not treated with SGLT2is, including in participants randomized to finerenone who were newly initiated on an SGLT2i during the trial.^43^ A separate prespecified secondary analysis of the 817 participants receiving finerenone and a SGLT2i showed a lifetime gain in event-free survival observed for a 65-year-old participant receiving finerenone plus a SGLT2i (3.1 years [95% CI 0.1–6.0, *P* = .04] vs 1.8 years [95% CI 0.5–3.1; *P* = .009] in a participant not taking a SGLT2i at baseline).^78^ In an analysis of three clinical trials, including FINEARTS-HF, combined use of finerenone and a SGLT2i reduced the risk of CV death or first worsening HF event by 31% (HR 0.69; 95% CI 0.59–0.81).^79^ For a 65-year-old patient, this translated to an additional 3.6 years (95% CI 2.0–5.2) free from an HF event or CV death.^79^

Ongoing trials^44,45,80^ will explore the efficacy and safety of finerenone in people hospitalized with acute decompensated HF and an EF ≥40% (REDEFINE-HF)^44^ and in combination with empagliflozin in people hospitalized (or recently discharged) for HF across the EF spectrum (CONFIRMATION-HF).^45^

The iCARDIO Alliance guidelines recommend finerenone to reduce HF hospitalizations in people with HFpEF, and in people with HFmrEF, estimated glomerular filtration rate >30 ml/min/1.73 m^2^, and potassium <5.0 mEq/L to reduce morbidity and mortality (*Table 1*).^19^ Guidelines in Japan recommend finerenone in people with symptomatic HF (Class IIa recommendation) to reduce the risk of CV death (HFpEF and HFmrEF) and HF hospitalization (HFpEF) or HF exacerbation events (HFmrEF).^5^ The European Society of Cardiology (ESC) and American College of Cardiology American Heart Association Heart Failure Society of America do not yet give a formal recommendation.

### Intravenous iron supplementation

Iron deficiency affects up to 50% of people with HF.^81^ ESC and iCardio Alliance guidelines recommend the use of intravenous iron supplementation with ferric carboxymaltose or ferric derisomaltose to correct iron deficiency in HFmrEF,^3,19^ while no recommendations are made for iron supplementation in HFpEF (*Table 1*).

## Unmet need in people with HFmrEF and HFpEF (with EF ≥40%)

Even with guideline-recommended therapy, residual risk for people with HFpEF and HFmrEF remains high.^36,38^ Several factors may contribute to the slower therapeutic development in individuals with HFpEF and HFmrEF.^18,82^ First, design choices in historical clinical trials regarding event rates and sample sizes have led most trials in HF to focus on people with HFrEF.^18^ It should also be noted that although EF is a continuum, ‘digit bias’, where clinicians report EF to numbers ending in ‘0’ or ‘5’, is common and may lead to misclassification of HF phenotypes.^83^

Second, people with HFpEF and HFmrEF represent a highly heterogeneous group with varying comorbidities/clinical profiles and a high risk of competing events that challenges trial design.^82^ There is also variability across clinical trials in terms of the inclusion criteria, leading to added heterogeneity across the different trial populations. Thus, HFpEF and HFmrEF may be considered a population comprising multiple ‘phenogroups’ and tailoring therapy to each individual's clinical profile may improve clinical outcomes in people with HFpEF and HFmrEF.^82^

Myocardial dysfunction in people with HFpEF could be a consequence of various comorbidities, including CV, obesity, kidney, metabolic, pulmonary, and geriatric conditions, and affects 5%–80% of this population.^82^ CV and kidney disease share similar pathophysiology and often coexist, making these conditions more difficult to manage. Cardio-kidney-metabolic disease is a complex disorder affecting the heart and kidneys that occurs in 40%–50% of people with HF, and CKD is frequently underdiagnosed in people with HF.^84,85^ The early assessment of kidney function in people presenting with HF is required to determine whether cardio-kidney disease is present and to inform appropriate management strategies to target both the heart and kidneys.^86,87^

New classes of therapies that target novel pathways implicated in the complex pathophysiology of HFpEF and HFmrEF and the comorbidities that contribute to their clinical presentation remain an unmet need. Dedicated trials are needed to fill key evidence gaps for existing and emerging HF therapies in people with HFpEF and HFmrEF.

Finally, non-pharmacologic factors are also important for optimizing the management of HFpEF and HFmrEF, but these are beyond the scope of this review. Briefly, age and sex are important demographic factors when assessing people with HFpEF, and may influence the burden associated with comorbidities of HF.^82,88^ Further establishing the clinical profiles of people with HFpEF and HFmrEF will help to provide timely diagnosis and appropriate treatment.^82^ The REVOLUTION-HF study found that 70% of people presenting to outpatient care with a combination of HF signs and/or symptoms and elevated N-terminal pro–B-type natriuretic peptide (NT-proBNP) levels were not diagnosed within the first year due to a lack of resources, resulting in a high risk of adverse outcomes.^89^

People with HFpEF or HFmrEF can also present with skeletal muscle dysfunction, which may contribute to exercise intolerance. Exercise training is an effective intervention for HFpEF; however, its effects on skeletal muscle function are poorly understood. Additional studies are needed to identify targeted therapies for people with HF presenting with skeletal muscle dysfunction.^90^ While beta blockers may have a potential benefit in HFpEF, they also induce chronotropic incompetence—an inability to increase heart rate during exercise—that is frequently observed in people with HFpEF and contributes to reduced exercise capacity,^91,92^ and improves following beta blocker withdrawal.^93^

Other cardiomyopathies, such as hypertrophic cardiomyopathy, Fabry disease, or cardiac amyloidosis, can present with a HFpEF-like phenotype.

## Emerging treatment options for people with EF ≥40%

**Figure 2:**
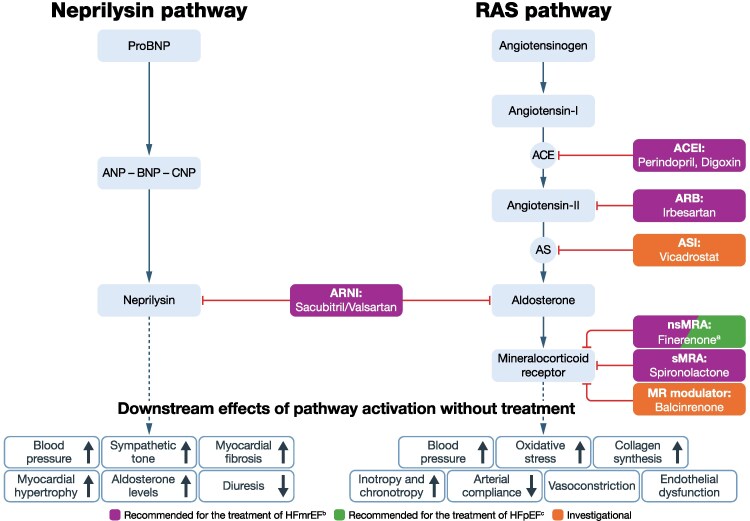
Pathway/receptor targets for current treatments and emerging therapies for HFpEF and HFmrEF: RAS and neprilysin pathways.^94–96^ ACC, American College of Cardiology; ACE, angiotensin-converting enzyme; ACEI, angiotensin-converting enzyme inhibitor; AHA, American Heart Association; ANP, atrial natriuretic peptide; ARB, angiotensin receptor blocker; ARNI, angiotensin receptor/neprilysin inhibitor; AS, aldosterone synthase; ASI, aldosterone synthase inhibitor; BNP, B-type natriuretic peptide; CNP, C-type natriuretic peptide; ESC, European Society of Cardiology; HFmrEF, heart failure with mildly reduced ejection fraction; HFpEF, heart failure with preserved ejection fraction; HFSA, Heart Failure Society of America; JCS, Japanese Circulation Society; JHFS, Japanese Heart Failure Society; MR, mineralocorticoid receptor; nsMRA, non-steroidal mineralocorticoid receptor antagonist; proBNP, pro–B-type natriuretic peptide; RAS, renin–angiotensin system; sMRA, steroidal mineralocorticoid receptor antagonist. ^a^Finerenone was approved for patients with HF and left ventricular EF ≥40% (i.e. HFpEF and HFmrEF) in the USA in July 2025;^75^ approval for patients with HF in Europe has not yet been granted. ^b^Based on ESC, AHA/ACC/HFSA or JCS/JHFS Class I or IIa recommendation, or iCARDIO Alliance ‘recommended’ or ‘strongly recommended’; ACEI or ARB recommended by iCARDIO Alliance; ARNI recommended by JCS/JHFS, and iCARDIO Alliance; finerenone (nsMRA) recommended by JCS/JHFS and iCARDIO Alliance; sMRA recommended by iCARDIO Alliance (see *Table 1* for details). ^c^Based on ESC, AHA/ACC/HFSA or JCS/JHFS Class I or Class IIa recommendation, or iCARDIO Alliance ‘recommended’ or ‘strongly recommended’; ARNI recommended for selected patients by JCS/JHFS and iCARDIO Alliance; finerenone (nsMRA) recommended by JCS/JHFS and iCARDIO Alliance (see *Table 1* for details)

**Figure 3:**
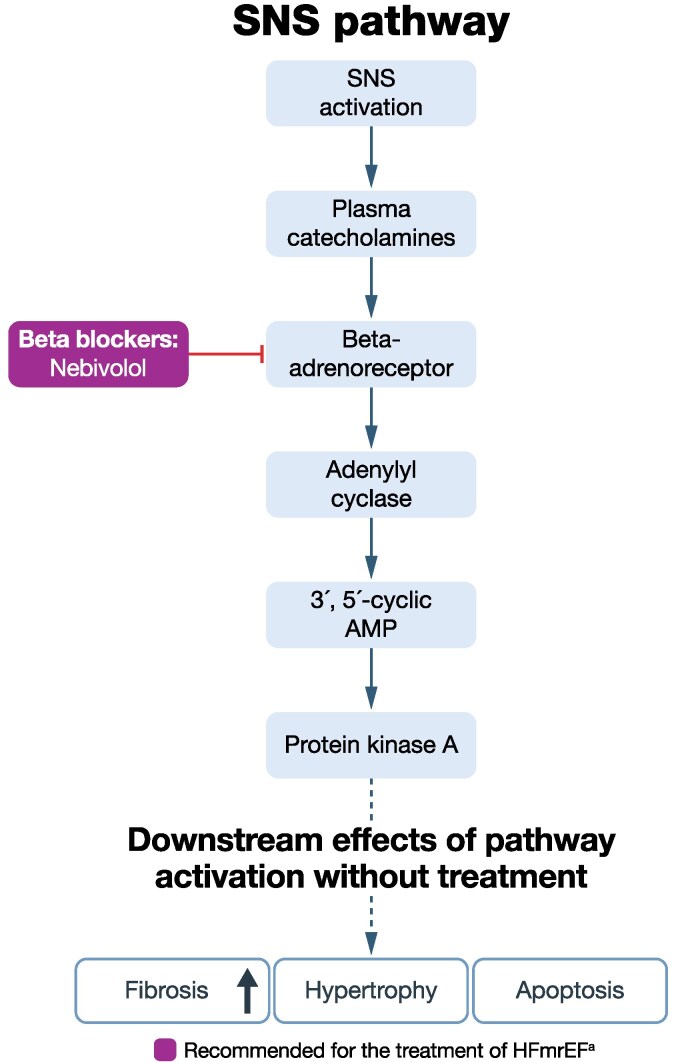
Pathway/receptor targets for current treatments and emerging therapies for HFpEF and HFmrEF: SNS pathway.^97,98^ ACC, American College of Cardiology; AHA, American Heart Association; AMP, adenosine 3′,5′-monophosphate; ESC, European Society of Cardiology; HFmrEF, heart failure with mildly reduced ejection fraction; HFpEF, heart failure with preserved ejection fraction; HFSA, Heart Failure Society of America; JCS, Japanese Circulation Society; JHFS, Japanese Heart Failure Society; SNS, sympathetic nervous system. ^a^Based on ESC, AHA/ACC/HFSA or JCS/JHFS Class I or Class IIa recommendation, or iCARDIO Alliance ‘recommended’ or ‘strongly recommended’; recommended by iCARDIO Alliance (see *Table 1* for details)

**Figure 4:**
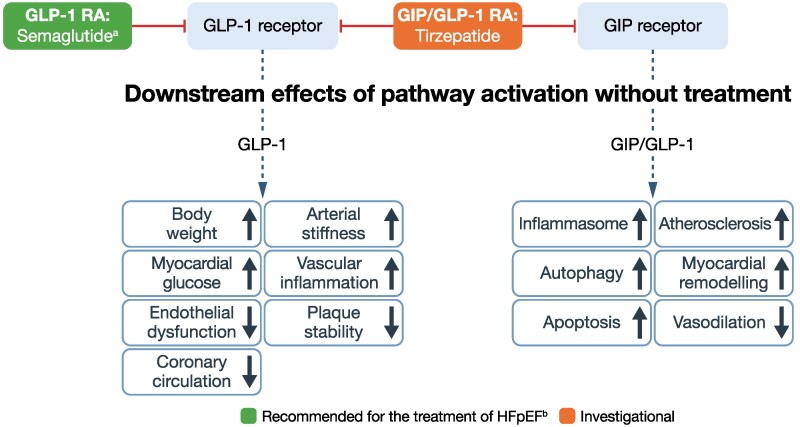
Pathway/receptor targets for current treatments and emerging therapies for HFpEF and HFmrEF: GLP-1 and GIP receptors.^99,100^ ACC, American College of Cardiology; AHA, American Heart Association; ESC, European Society of Cardiology; GIP, glucose-dependent insulinotropic polypeptide; GLP-1, glucagon-like peptide 1; GLP-1 RA, glucagon-like peptide 1 receptor antagonist; HFmrEF, heart failure with mildly reduced ejection fraction; HFpEF, heart failure with preserved ejection fraction; HFSA, Heart Failure Society of America; JCS, Japanese Circulation Society; JHFS, Japanese Heart Failure Society. ^a^Prescribing information for semaglutide does not include HFpEF as an indication;^101^ however, global guidelines recommend its use in people with HFpEF and obesity.^19^  ^a^Based on ESC, AHA/ACC/HFSA or JCS/JHFS Class I or Class IIa recommendation or above, or iCARDIO Alliance ‘recommended’ or ‘strongly recommended’: recommended by iCARDIO Alliance for people with obesity and HFpEF (see *Table 1* for details)

**Figure 5:**
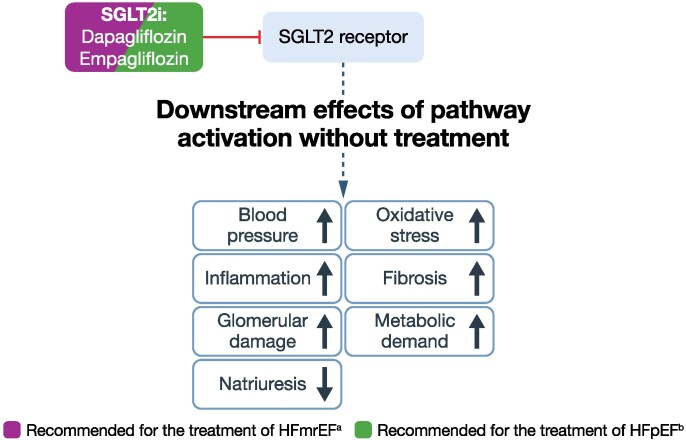
Pathway/receptor targets for current treatments and emerging therapies for HFpEF and HFmrEF: SGLT2 receptor.^99^ ACC, American College of Cardiology; AHA, American Heart Association; ESC, European Society of Cardiology; HFmrEF, heart failure with mildly reduced ejection fraction; HFpEF, heart failure with preserved ejection fraction; HFSA, Heart Failure Society of America; JCS, Japanese Circulation Society; JHFS, Japanese Heart Failure Society; SGLT2, sodium–glucose cotransporter 2; SGLT2i, sodium–glucose cotransporter 2 inhibitor. ^a^Based on ESC, AHA/ACC/HFSA or JCS/JHFS Class I or Class IIa recommendation or higher, or iCARDIO Alliance ‘recommended’ or ‘strongly recommended’: recommended by ESC, AHA/ACC/HFSA, JCS/JHFS, and iCARDIO Alliance (see *Table 1* for details). ^b^Based on ESC, AHA/ACC/HFSA or JCS/JHFS Class I or Class IIa recommendation, or iCARDIO Alliance ‘recommended’ or ‘strongly recommended’; recommended by ESC, AHA/ACC/HFSA, JCS/JHFS, and iCARDIO Alliance (see *Table 1* for details)

Due to the residual risk in people with HFpEF and HFmrEF driven by the heterogeneity of these conditions, the burden of comorbidities, and the persistent risk of adverse outcomes, several emerging treatments are being investigated for their potential benefits in this population. Treatment targets of emerging therapies relative to current therapies are presented in *Figures 2–5*.^19,75,94-101^

### MR modulators

Balcinrenone, a selective MR modulator, is being investigated for its efficacy and safety in people with HF (*Table 3*).^102,103,110^ In the Phase 2b study MIRACLE, balcinrenone in combination with dapagliflozin in people with HF (EF <60%, mean EF 46%) and CKD did not improve the primary endpoint of geometric mean percentage change in urine albumin-to-creatinine ratio from baseline to Week 12 compared with dapagliflozin alone.^102^ The ongoing BALANCED-HF Phase 3 trial will further evaluate the role of balcinrenone in combination with dapagliflozin in people with HF with a recent HF event and impaired kidney function, and is recruiting people with HF across the EF spectrum.^103^ A positive effect of balcinrenone, irrespective of EF and with an acceptable safety profile, will support the use of balcinrenone in people with HFpEF and HFmrEF.

**Table 3:** Emerging treatment options in people with HFpEF and HFmrEF

Trial	Drug	Trial population and key inclusion criteria	Patient profile at baseline (mean, unless stated otherwise)	Key outcomes or results (if available; vs placebo)
MR modulator				
MIRACLE^102^	Balcinrenone plus dapagliflozin vs dapagliflozin	HF and CKD (*N* = 133) EF <60% eGFR ≥30 to ≤60 ml/min/1.73 m^2^ UACR ≥30 to *<*3000 mg/g Aged ≥21 years Elevated NT-proBNP^a^	Age: 72.3 years BMI: NR Diabetes (T2D): 65.4% EF: 46.0% eGFR: 41.7 ml/min/1.73 m^2^	Primary outcome—relative change in UACR from baseline to Week 12 (95% CI): Balcinrenone 15 mg + dapagliflozin 10 mg, –33.6% (–62.5 to 17.6); *P* = .1588 Balcinrenone 50 mg + dapagliflozin 10 mg, –11.8% (–52.2 to 62.6); *P* = .6846 Balcinrenone 150 mg + dapagliflozin 10 mg, –36.1% (–64.9 to 16.1); *P* = .1398
BALANCED-HF^103^	Balcinrenone plus dapagliflozin vs dapagliflozin	HF with recent HF event, (estimated *N* = 4800) eGFR 20 to <60 ml/min/1.73 m^2^ Aged ≥18 years Elevated NT-proBNP^a^ HF hospitalization or urgent visit within ≤6 months	NA—trial ongoing	Primary outcome: Composite of CV death, hospitalization for HF, or HF event without hospitalization Secondary outcomes: Occurrence of primary outcome components Hierarchical composite endpoint of death from any cause, total HF events, and change in KCCQ-TSS from baseline to 24 weeks post-randomization
ASI				
EASi-HF^104^	Vicadrostat plus empagliflozin vs empagliflozin	HFpEF and HFmrEF (estimated *N* = 6000) EF ≥40% Aged ≥18 years Elevated NT-proBNP^b^ Current diuretic therapy or HF hospitalization within ≤6 months	NA—trial ongoing	Primary outcome: Composite of time to CV death, hospitalization for HF, or urgent HF visit vs empagliflozin alone Secondary outcomes: Time to first event of CV death or hospitalization for HF Occurrence of hospitalization for HF (first and recurrent) Change from baseline in KCCQ-TSS and KCCQ-CSS at Week 32
GLP-1 RA				
STEP-HFpEF^105^	Semaglutide	HFpEF and obesity (*N* = 529) EF ≥45% Aged ≥18 years BMI ≥30 kg/m^2^ Elevated LV filling pressure or elevated NT-proBNP^c^ or HF hospitalization within ≤12 months plus current diuretic therapy	Age (median): 69.0 years BMI (median): 37.0 kg/m^2^ EF 45%–49%: 16.1% EF ≥50%: 83.9% CKD: NR	Primary outcome (mean change in KCCQ-CSS from baseline to Week 52): Difference vs placebo 7.8 (95% CI 4.8–10.9), *P* < .001 Secondary outcome (hierarchical composite including death, HF events, and differences in the change in KCCQ-CSS and 6MWD): Win ratio: 1.72 (95% CI 1.37–2.15); *P* < .001
STEP-HFpEF DM^106^	Semaglutide	HFpEF, obesity, and T2D (*N* = 616) EF ≥45% Aged ≥18 years BMI ≥30 kg/m^2^ Elevated LV filling pressure or elevated NT-proBNP^c^ plus echocardiographic abnormalities or HF hospitalization within ≤12 months plus ongoing diuretic therapy	Age (median): 69.0 years BMI (median): 36.9 kg/m^2^ Diabetes (T2D): 100% EF 45%–49%: 17.2% EF ≥50%: 82.8% eGFR (median): 69.2 ml/min/1.73 m^2^	Primary outcome (mean change in KCCQ-CSS from baseline to Week 52): Difference vs placebo 7.3 (95% CI 4.1–10.4), *P* < .001 Secondary outcome (hierarchical composite including death, HF events, and differences in the change in KCCQ-CSS and 6MWD): Win ratio: 1.58 (95% CI 1.29–1.94); *P* < .001
Pooled STEP-HFpEF and STEP-HFpEF DM analysis^107,108^	Semaglutide	HFpEF and obesity (*N* = 1145) EF ≥45% Aged ≥18 years BMI ≥30 kg/m^2^ Elevated LV filling pressure or elevated NT-proBNP^c^ Echocardiographic abnormalities or HF hospitalization within ≤12 months plus ongoing diuretic therapy	Age (median): 69.5 years BMI (median): 37.0 kg/m^2^ EF 45%–49%: 16.6% EF ≥50%: 83.4% CKD: NR	Secondary outcome (hierarchical composite including death, HF events, and differences in the change in KCCQ-CSS and 6MWD): Win ratio: 1.65 (95% CI 1.42–1.91); *P* < .0001 Change in NT-proBNP at 52 weeks: Estimated treatment ratio: 0.82 (95% CI 0.74–0.91); *P* = .0002
Dual GIP/GLP-1 RA				
SUMMIT^109^	Tirzepatide	HFpEF and obesity (*N* = 731) EF >50% Aged ≥40 years BMI ≥30 kg/m^2^ Elevated NT-proBNP^d^ left atrial enlargement, or elevated LV filling pressure HF decompensation within ≤12 months or eGFR <70 ml/min/1.73 m^2^	Age: 65.2 years BMI: 38.2 kg/m^2^ Diabetes (T2D): 48.2% EF <60%: 39.1% EF ≥60%: 60.9% CKD (eGFR <60 ml/min/1.73 m^2^): 45.8%	Primary outcome—composite of CV death or worsening HF: HR: 0.62 (95% CI 0.41–0.95); *P* = .026 EF <60%: HR: 0.60 (95% CI 0.33–1.09), EF ≥60%: HR: 0.60 (95% CI 0.33–1.09); interaction *P* = NR Primary outcome—change at 52 weeks in KCCQ-CSS: Difference 6.9 (95% CI 3.3–10.6); *P* < .001 EF <60%: difference 5.30 (95% CI –0.12 to 10.73), EF ≥60%: difference 8.08 (95% CI 3.97–12.19); interaction *P* = NR Receiving MRA at baseline: Yes: HR: 0.58 (95% CI 0.31–1.09) No: HR: 0.63 (95% CI 0.36–1.10)
Correction of iron deficiency				
FAIR-HFpEF^81^	Ferric carboxymaltose	HFpEF iron deficiency (*N* = 39) EF ≥45% Ferritin <100 ng/ml or ferritin 100–299 ng/ml plus transferrin saturation <20% Aged ≥18 years HF hospitalization within≤12 months Elevated NT-proBNP or BNP^e^	Age (median): 80 years BMI (median): 29.4 kg/m^2^ Diabetes: 46.1% EF (median): 55%	Difference vs placebo in change in 6MWD at Week 24: 49 m (95% CI 5–93); *P* = .029

6MWD, 6-minute walking distance; AF, atrial fibrillation; ASI, aldosterone synthase inhibitor; BMI, body mass index; BNP, B-type natriuretic peptide; CI, confidence interval; CKD, chronic kidney disease; CV, cardiovascular; EF, ejection fraction; eGFR, estimated glomerular filtration rate; GIP, glucose-dependent insulinotropic polypeptide; GLP-1 RA, glucagon-like peptide-1 receptor agonist; HF, heart failure; HFmrEF, heart failure with mildly reduced ejection fraction; HFpEF, heart failure with preserved ejection fraction; HR, hazard ratio; KCCQ-CSS, Kansas City Cardiomyopathy Questionnaire Clinical Summary Score; KCCQ-TSS, Kansas City Cardiomyopathy Questionnaire Total Symptom Score; LV, left ventricular; MR, mineralocorticoid receptor; NA, not available; NR, not reported; nsMRA, non-steroidal mineralocorticoid receptor antagonist; NT-proBNP, N-terminal pro–B-type natriuretic peptide; RR, risk ratio; sMRA, steroidal mineralocorticoid receptor antagonist; T2D, type 2 diabetes; UACR, urine albumin-to-creatinine ratio.

^a^NT-proBNP ≥300 pg/ml (≥600 pg/ml if in AF).

^b^NT-proBNP ≥300 pg/ml (≥900 pg/ml if in AF), ≥220 pg/ml (≥660 pg/ml if in AF), or ≥125 pg/ml (≥375 pg/ml if in AF), for participants with BMI <27, 27–<35, or ≥35 kg/m^2^, respectively.

^c^NT-proBNP ≥220 pg/ml (≥660 pg/ml if in AF) or ≥125 pg/ml (≥375 pg/ml if in AF), for participants with BMI <35 or ≥35 kg/m^2^, respectively.

^d^NT-proBNP >200 pg/ml (>600 pg/ml if in AF).

^e^NT-proBNP >300 pg/ml or BNP >100 pg/ml (>600 pg/ml or >200 pg/ml, respectively, if in AF).

### Aldosterone synthase inhibitors (ASIs)

ASIs are an emerging treatment class that may provide an alternative method to target the effects of excess aldosterone observed in CV disease pathophysiology,^94^ and eliminate off-target effects of excess aldosterone frequently observed with sMRA and nsMRAs.^111^ EASI-HFpEF will evaluate the efficacy and safety of the ASI vicadrostat plus empagliflozin compared with placebo and empagliflozin in approximately 6000 participants with symptomatic HFpEF and HFmrEF not on sMRAs or an nsMRA (*Table 3*).^104^ A positive finding in this study will support the use of ASIs in people with HFpEF or HFmrEF.

### Glucagon-like peptide-1 receptor agonists (GLP-1 RAs) and glucose-dependent insulinotropic polypeptide receptor agonists (GIP RAs)

The effects of GLP-1 RAs on atherosclerotic CV disease in T2D, kidney outcomes, and obesity are well established.^112–114^ In addition, a meta-analysis indicated that treatment with a GLP-1 RA may prevent new-onset HF and mortality in people with T2D without HF.^115^

Two recently published trials have demonstrated the benefit of the GLP-1 RA semaglutide in people with obesity-related HF and an EF ≥45%. In the STEP-HFpEF trial, semaglutide reduced the secondary hierarchical composite endpoint, which included death and HF events, and improved KCCQ-CSS and 6-min walking distance compared with placebo (*Table 3*).^105^ The STEP-HFpEF DM study demonstrated similar effects on the same hierarchical composite endpoint in people with obesity-related HF (EF ≥45%) and T2D (*Table 3*).^106^ A prespecified analysis of pooled data from STEP-HFpEF and STEP-HFpEF DM confirmed the results of the individual studies with regard to the hierarchical endpoint (*Table 3*).^107^ Semaglutide reduced NT-proBNP at 52 weeks compared with placebo (*Table 3*)^108^ and was also associated with a reduction in loop diuretic use and dose compared with placebo.^116^ Although these trials were double-blind studies, the side effects and the body weight loss associated with GLP-1 RAs may undermine the blinded nature of the study, and the results should be interpreted with caution.

Safety results from the pooled analysis demonstrated that semaglutide was well tolerated in people with obesity-related HFpEF with or without T2D.^107^ Although gastrointestinal events leading to discontinuation were higher with semaglutide than placebo, the overall frequencies of gastrointestinal serious AEs were similar in both groups.^107^ Current treatment recommendations for GLP-1 RAs based on findings of studies with semaglutide are limited to people with obesity.

Tirzepatide is a long-acting dual agonist of GIP and GLP-1 (*Figure 2*), with a greater affinity towards the GIP receptor. This imbalance in agonism is considered important for its efficacy as dose escalation of GLP-1 RA can be limited due to gastrointestinal AEs. In SUMMIT, treatment with tirzepatide reduced the risk of the two co-primary endpoints, the composite of CV death or worsening HF (*Table 3*), and improved health status (KCCQ-CSS between-group difference: 6.9, *P* < .001).^109^ This benefit was observed irrespective of MRA use at baseline (*Table 3*).^109^ Although gastrointestinal symptoms were common with tirzepatide, they tended to dissipate over time and led to treatment discontinuation in only 4% of participants; the frequencies of serious AEs were similar between the tirzepatide and placebo groups.^109^ A cardiac magnetic resonance substudy of SUMMIT (*n* = 106) demonstrated that tirzepatide decreased left ventricular mass compared with placebo, and that the change in left ventricular mass was correlated with body weight loss.^117^ These findings suggest that changes in cardiac structure and a reduction in pro-inflammatory paracardiac adipose tissue may contribute to the decrease in HF events observed with tirzepatide in the main SUMMIT trial.^117^

The studies described above provide support for further evaluation of the efficacy of GLP-1 RAs and GIP/GLP-1 RAs in these populations.

### Intravenous iron supplementation

While not currently recommended in the guidelines for HFpEF, results from the FAIR-HFpEF study suggest there may be potential benefits of ferric carboxymaltose treatment in participants with HFpEF and iron deficiency.^81^

### New treatment approaches: combination therapy and precision medicine

Expert opinion increasingly supports the benefits of simultaneous or rapid sequence initiation of combination therapy for people with HFpEF and HFmrEF.^118,119^ To this end, recently, a three-pillared approach to treatment of HFpEF and HFmrEF was proposed, incorporating simultaneous/rapid sequence initiation of SGLT2is, an nsMRA, and GLP-1 RAs in combination.^119^ However, evidence to support this approach is currently lacking. Another potential three-pronged therapeutic strategy involves the use of an SGLT2i, an nsMRA, and an ARNI. In a recent analysis of data from three clinical trials in people with HFmrHF and HFpEF (DELIVER, FINEARTS HF, and PARAGON-HF), this combination reduced the risk of cardiovascular death or first worsening HF event by 39% in those with left ventricular EF <60% (HR 0.61; 95% CI 0.48–0.77).^79^

Furthermore, the heterogenous nature of HFpEF and HFmrEF due to the variable pathologic drivers, risk factors, and underlying aetiologies suggests that a shift toward phenotype-specific treatment strategies is warranted.^120,121^ Despite the heterogeneity between patients, the variability of clinical trial inclusion criteria, and a limited understanding of risk factors and predictors of HFpEF and HFmrEF, recent studies have proposed three broadly similar phenotypic clusters for HFpEF based on demographic and clinical characteristics.^120–122^ Specifically, the HFpEF phenotype clusters are based on age, comorbidity type, and burden of comorbid disease.^122^ Patients in phenotype 1 tend to be younger and have fewer comorbidities, those in phenotype 2 are older and are more likely to have comorbid atrial fibrillation and cardiorenal disease, while patients in phenotype 3 are of intermediate age with high comorbid disease burden including higher rates of T2D and obesity.^122^

Personalized approaches to treatment of certain subgroups of people based on comorbidities may be considered.^118,119^ For example, in patients with comorbid CKD, RASis or ARNIs for HFpEF and HFmrEF can be initiated as well as SGLT2is and an nsMRA.^118^ In addition, in a phenotype-guided treatment approach, GLP-1 RAs should be initiated in combination with SGLT2is and an nsMRA in patients with HFpEF or HFmrEF and comorbid CKD and T2D or obesity.^118^

Although new research is needed to further understand HFpEF and HFmrEF, especially given their complexity and associated comorbidities, continuing to explore optimal treatment sequencing and precision medication approaches will help with addressing the unmet medical needs and therapeutic gaps for patients with EF >40%.

### Additional combination treatment considerations

As effective treatment options for patients with HFpEF and HFmrEF expand, guideline-directed treatments will likely evolve to include simultaneous initiation of combinations of drugs, similar to the current treatment approach for HFrEF. Polypharmacy will necessitate increased monitoring for electrolytes, blood pressure, and kidney function, and additional considerations related to the safety, access, and cost-effectiveness of combination therapy must be taken into account. The safety and tolerability of combination regimens for HFpEF and HFmrEF will need to be considered based on clinical trial findings alongside individual patient factors based on their comorbidities. Such an approach is fundamental to precision medicine. Polypharmacy costs may be a barrier for some patient populations; however, costs for healthcare systems may be lower if the number of HF hospitalizations is reduced, as has been observed with combination treatment in HFrEF.^118^

## Conclusion

Despite guideline-directed therapy,^18,59,82^ an unmet need for effective pharmacologic treatment options remains for people with HFpEF and HFmrEF. Moreover, treatment is further complicated by the heterogeneity of comorbidities and clinical profiles in these populations, as also seen across clinical trials, which can significantly influence outcomes.

SGLT2is and the nsMRA finerenone have shown efficacy in improving outcomes for individuals with HFpEF and HFmrEF and are currently being further investigated in ongoing studies. Additionally, multiple trials are underway to explore new classes of treatments for HFpEF and HFmrEF, aiming to expand available therapeutic options.^44,45,80,103,104^ Further improvement in outcomes may also be achieved by tailoring treatment to specific phenotypes, such as obesity and CKD.^118,119^

Ongoing studies addressing key knowledge gaps in the management of HFpEF and HFmrEF, along with the integration of emerging and existing therapies into clinical guidelines, are essential for advancing treatment strategies, improving outcomes, and addressing the unmet therapeutic needs for people with HFpEF and HFmrEF.

## Declarations

### Data Availability

Availability of the data underlying this publication will be determined according to Bayer’s commitment to the EFPIA/PhRMA ‘Principles for responsible clinical trial data sharing’. This pertains to scope, timepoint, and process of data access.

As such, Bayer commits to sharing upon request from qualified scientific and medical researchers’ patient-level clinical trial data, study-level clinical trial data, and protocols from clinical trials in patients for medicines and indications approved in the United States (US) and European Union (EU) as necessary for conducting legitimate research. This applies to data on new medicines and indications that have been approved by the EU and US regulatory agencies on or after 01 January 2014.

Interested researchers can use www.vivli.org to request access to anonymized patient-level data and supporting documents from clinical studies to conduct further research that can help advance medical science or improve patient care. Information on the Bayer criteria for listing studies and other relevant information is provided in the member section of the portal.

Data access will be granted to anonymized patient-level data, protocols, and clinical study reports after approval by an independent scientific review panel. Bayer is not involved in the decisions made by the independent review panel. Bayer will take all necessary measures to ensure that patient privacy is safeguarded.

## References

[1] Bozkurt B, Coats AJS, Tsutsui H, Abdelhamid CM, Adamopoulos S, Albert N, et al Universal definition and classification of heart failure: a report of the Heart Failure Society of America, Heart Failure Association of the European Society of Cardiology, Japanese Heart Failure Society and Writing Committee of the Universal Definition of Heart Failure: Endorsed by the Canadian Heart Failure Society, Heart Failure Association of India, Cardiac Society of Australia and New Zealand, and Chinese Heart Failure Association. Eur J Heart Fail 2021;23:352–80. 10.1002/ejhf.2115 33605000

[2] Heidenreich PA, Bozkurt B, Aguilar D, Allen LA, Byun JJ, Colvin MM, et al 2022 AHA/ACC/HFSA guideline for the management of heart failure: a report of the American College of Cardiology/American Heart Association Joint Committee on Clinical Practice Guidelines. Circulation 2022;145:e895–1032. 10.1161/CIR.0000000000001063 35363499

[3] McDonagh TA, Metra M, Adamo M, Gardner RS, Baumbach A, Böhm M, et al 2023 focused update of the 2021 ESC guidelines for the diagnosis and treatment of acute and chronic heart failure. Eur Heart J 2023;44:3627–39. 10.1093/eurheartj/ehad195 37622666

[4] McDonagh TA, Metra M, Adamo M, Gardner RS, Baumbach A, Böhm M, et al 2021 ESC guidelines for the diagnosis and treatment of acute and chronic heart failure. Eur Heart J 2021;42:3599–726. 10.1093/eurheartj/ehab368 34447992

[5] Kitai T, Kohsaka S, Kato T, Kato E, Sato K, Teramoto K, et al JCS/JHFS 2025 guideline on diagnosis and treatment of heart failure. Circ J 2025;89:1278–444. 10.1253/circj.CJ-25-0002 40159241

[6] Tan C, Dinh D, Brennan A, Hare DL, Kaye D, Lefkovits J, et al Characteristics and clinical outcomes in patients with heart failure with preserved ejection fraction compared to heart failure with reduced ejection fraction: insights from the VCOR heart failure snapshot. Heart Lung Circ 2022;31:623–8. 10.1016/j.hlc.2021.09.019 34742643

[7] Savarese G, Stolfo D, Sinagra G, Lund LH. Heart failure with mid-range or mildly reduced ejection fraction. Nat Rev Cardiol 2022;19:100–16. 10.1038/s41569-021-00605-5 34489589 PMC8420965

[8] Palazzuoli A, Beltrami M. Are HFpEF and HFmrEF so different? The need to understand distinct phenotypes. Front Cardiovasc Med 2021;8:676658. 10.3389/fcvm.2021.676658 34095263 PMC8175976

[9] Savarese G, Becher PM, Lund LH, Seferovic P, Rosano GMC, Coats AJS. Global burden of heart failure: a comprehensive and updated review of epidemiology. Cardiovasc Res 2023;118:3272–87. 10.1093/cvr/cvac013 35150240

[10] Hassan L, Efremov L, Grosskopf A, Kartschmit N, Medenwald D, Schott A, et al Cardiovascular risk factors, living and ageing in Halle: the CARLA study. Eur J Epidemiol 2022;37:103–16. 10.1007/s10654-021-00824-7 34978665 PMC8791893

[11] Borlaug BA, Sharma K, Shah SJ, Ho JE. Heart failure with preserved ejection fraction: JACC scientific statement. J Am Coll Cardiol 2023;81:1810–34. 10.1016/j.jacc.2023.01.049 37137592

[12] Tsao CW, Lyass A, Enserro D, Larson MG, Ho JE, Kizer JR, et al Temporal trends in the incidence of and mortality associated with heart failure with preserved and reduced ejection fraction. JACC Heart Fail 2018;6:678–85. 10.1016/j.jchf.2018.03.006 30007560 PMC6076350

[13] Kapelios CJ, Shahim B, Lund LH, Savarese G. Epidemiology, clinical characteristics and cause-specific outcomes in heart failure with preserved ejection fraction. Card Fail Rev 2023;9:e14. 10.15420/cfr.2023.03 38020671 PMC10680134

[14] Settergren C, Benson L, Shahim A, Dahlstrom U, Thorvaldsen T, Savarese G, et al Cause-specific death in heart failure across the ejection fraction spectrum: a comprehensive assessment of over 100 000 patients in the Swedish Heart Failure Registry. Eur J Heart Fail 2024;26:1150–9. 10.1002/ejhf.3230 38606645

[15] Chioncel O, Lainscak M, Seferovic PM, Anker SD, Crespo-Leiro MG, Harjola VP, et al Epidemiology and one-year outcomes in patients with chronic heart failure and preserved, mid-range and reduced ejection fraction: an analysis of the ESC Heart Failure Long-Term Registry. Eur J Heart Fail 2017;19:1574–85. 10.1002/ejhf.813 28386917

[16] Lam CSP, Wood R, Vaduganathan M, Bueno H, Chin A, Luporini Saraiva G, et al Contemporary economic burden in a real-world heart failure population with commercial and Medicare supplemental plans. Clin Cardiol 2021;44:646–55. 10.1002/clc.23585 33704817 PMC8119853

[17] Bundgaard JS, Mogensen UM, Christensen S, Ploug U, Rorth R, Ibsen R, et al Healthcare cost variation in patients with heart failure: a nationwide study. Public Health 2022;207:88–93. 10.1016/j.puhe.2022.03.019 35594807

[18] Pfeffer MA, Shah AM, Borlaug BA. Heart failure with preserved ejection fraction in perspective. Circ Res 2019;124:1598–617. 10.1161/circresaha.119.313572 31120821 PMC6534165

[19] Chopra V, Khan MS, Abdelhamid M, Abraham WT, Amire A, Anker SD, et al iCARDIO alliance global implementation guidelines on heart failure 2025. Heart Lung Circ 2025;34:e55–82. 10.1016/j.hlc.2025.05.094 40533340

[20] Cleland JG, Tendera M, Adamus J, Freemantle N, Polonski L, Taylor J. The perindopril in elderly people with chronic heart failure (PEP-CHF) study. Eur Heart J 2006;27:2338–45. 10.1093/eurheartj/ehl250 16963472

[21] Yusuf S, Pfeffer MA, Swedberg K, Granger CB, Held P, McMurray JJ, et al Effects of candesartan in patients with chronic heart failure and preserved left-ventricular ejection fraction: the CHARM-preserved trial. Lancet 2003;362:777–81. 10.1016/s0140-6736(03)14285-7 13678871

[22] Massie BM, Carson PE, McMurray JJ, Komajda M, McKelvie R, Zile MR, et al Irbesartan in patients with heart failure and preserved ejection fraction. N Engl J Med 2008;359:2456–67. 10.1056/NEJMoa0805450 19001508

[23] Kao DP, Lewsey JD, Anand IS, Massie BM, Zile MR, Carson PE, et al Characterization of subgroups of heart failure patients with preserved ejection fraction with possible implications for prognosis and treatment response. Eur J Heart Fail 2015;17:925–35. 10.1002/ejhf.327 26250359 PMC4654630

[24] Ahmed A, Rich MW, Fleg JL, Zile MR, Young JB, Kitzman DW, et al Effects of digoxin on morbidity and mortality in diastolic heart failure: the ancillary digitalis investigation group trial. Circulation 2006;114:397–403. 10.1161/circulationaha.106.628347 16864724 PMC2628473

[25] Lund LH, Claggett B, Liu J, Lam CS, Jhund PS, Rosano GM, et al Heart failure with mid-range ejection fraction in CHARM: characteristics, outcomes and effect of candesartan across the entire ejection fraction spectrum. Eur J Heart Fail 2018;20:1230–9. 10.1002/ejhf.1149 29431256

[26] Solomon SD, McMurray JJV, Anand IS, Ge J, Lam CSP, Maggioni AP, et al Angiotensin-neprilysin inhibition in heart failure with preserved ejection fraction. N Engl J Med 2019;381:1609–20. 10.1056/NEJMoa1908655 31475794

[27] Solomon SD, Vaduganathan M, LC B, Packer M, Zile M, Swedberg K, et al Sacubitril/valsartan across the spectrum of ejection fraction in heart failure. Circulation 2020;141:352–61. 10.1161/circulationaha.119.044586 31736342

[28] Mentz RJ, Ward JH, Hernandez AF, Lepage S, Morrow DA, Sarwat S, et al Angiotensin-neprilysin inhibition in patients with mildly reduced or preserved ejection fraction and worsening heart failure. J Am Coll Cardiol 2023;82:1–12. 10.1016/j.jacc.2023.04.019 37212758

[29] Pitt B, Pfeffer MA, Assmann SF, Boineau R, Anand IS, Claggett B, et al Spironolactone for heart failure with preserved ejection fraction. N Engl J Med 2014;370:1383–92. 10.1056/NEJMoa1313731 24716680

[30] Solomon SD, Claggett B, Lewis EF, Desai A, Anand I, Sweitzer NK, et al Influence of ejection fraction on outcomes and efficacy of spironolactone in patients with heart failure with preserved ejection fraction. Eur Heart J 2016;37:455–62. 10.1093/eurheartj/ehv464 26374849 PMC4751235

[31] Pfeffer MA, Claggett B, Assmann SF, Boineau R, Anand IS, Clausell N, et al Regional variation in patients and outcomes in the Treatment of Preserved Cardiac Function Heart Failure With an Aldosterone Antagonist (TOPCAT) trial. Circulation 2015;131:34–42. 10.1161/CIRCULATIONAHA.114.013255 25406305

[32] Cohen JB, Schrauben SJ, Zhao L, Basso MD, Cvijic ME, Li Z, et al Clinical phenogroups in heart failure with preserved ejection fraction: detailed phenotypes, prognosis, and response to spironolactone. JACC Heart Fail 2020;8:172–84. 10.1016/j.jchf.2019.09.009 31926856 PMC7058514

[33] ClinicalTrials.gov . *Spironolactone in the Treatment of Heart Failure (SPIRIT-HF)*. 2024. https://clinicaltrials.gov/study/NCT04727073 (24 February 2026, date last accessed).

[34] Lund LH, James S, DeVore AD, Anstrom KJ, Fudim M, Aaronson KD, et al The spironolactone initiation registry randomized interventional trial in heart failure with preserved ejection fraction (SPIRRIT-HFpEF): rationale and design. Eur J Heart Fail 2024;26:2453–63. 10.1002/ejhf.3453 39282788 PMC11659483

[35] Cleland JGF, Bunting KV, Flather MD, Altman DG, Holmes J, Coats AJS, et al Beta-blockers for heart failure with reduced, mid-range, and preserved ejection fraction: an individual patient-level analysis of double-blind randomized trials. Eur Heart J 2018;39:26–35. 10.1093/eurheartj/ehx564 29040525 PMC5837435

[36] Anker SD, Butler J, Filippatos G, Ferreira JP, Bocchi E, Bohm M, et al Empagliflozin in heart failure with a preserved ejection fraction. N Engl J Med 2021;385:1451–61. 10.1056/NEJMoa2107038 34449189

[37] Sharma A, Ferreira JP, Zannad F, Pocock SJ, Filippatos G, Pfarr E, et al Cardiac and kidney benefits of empagliflozin in heart failure across the spectrum of kidney function: insights from the EMPEROR-Preserved trial. Eur J Heart Fail 2023;25:1337–48. 10.1002/ejhf.2857 37062851

[38] Solomon SD, McMurray JJV, Claggett B, de Boer RA, DeMets D, Hernandez AF, et al Dapagliflozin in heart failure with mildly reduced or preserved ejection fraction. N Engl J Med 2022;387:1089–98. 10.1056/NEJMoa2206286 36027570

[39] Nassif ME, Windsor SL, Borlaug BA, Kitzman DW, Shah SJ, Tang F, et al The SGLT2 inhibitor dapagliflozin in heart failure with preserved ejection fraction: a multicenter randomized trial. Nat Med 2021;27:1954–60. 10.1038/s41591-021-01536-x 34711976 PMC8604725

[40] Solomon SD, McMurray JJV, Vaduganathan M, Claggett B, Jhund P, Desai A, et al Finerenone in heart failure with mildly reduced or preserved ejection fraction. N Engl J Med 2024;391:1475–85. 10.1056/NEJMoa2407107 39225278

[41] Solomon SD, Ostrominski JW, Vaduganathan M, Claggett B, Jhund PS, Desai AS, et al Baseline characteristics of patients with heart failure with mildly reduced or preserved ejection fraction: the FINEARTS-HF trial. Eur J Heart Fail 2024;26:1334–46. 10.1002/ejhf.3266 38733212

[42] Yang M, Henderson AD, Talebi A, Atherton JJ, Chiang CE, Chopra V, et al Effect of finerenone on the KCCQ in patients with HFmrEF/HFpEF: a prespecified analysis of FINEARTS-HF. J Am Coll Cardiol 2025;85:120–36. 10.1016/j.jacc.2024.09.023 39520455

[43] Vaduganathan M, Claggett BL, Kulac IJ, Miao ZM, Desai AS, Jhund PS, et al Effects of the nonsteroidal MRA finerenone with and without concomitant SGLT2 inhibitor use in heart failure. Circulation 2025;151:149–58. 10.1161/CIRCULATIONAHA.124.072055 39340828 PMC11732259

[44] ClinicalTrials.gov . *A Study to Determine the Efficacy and Safety of Finerenone on Morbidity and Mortality Among Hospitalized Heart Failure Patients (REDEFINE-HF)*. 2024. https://classic.clinicaltrials.gov/ct2/show/NCT06008197 (24 February 2026, date last accessed).

[45] ClinicalTrials.gov . *A Study to Determine the Efficacy and Safety of Finerenone and SGLT2i in Combination in Hospitalized Patients With Heart Failure (CONFIRMATION-HF) (CONFIRMATION)*. 2024. https://classic.clinicaltrials.gov/ct2/show/NCT06024746 (24 February 2026 date last accessed).

[46] Hartupee J, Mann DL. Neurohormonal activation in heart failure with reduced ejection fraction. Nat Rev Cardiol 2017;14:30–8. 10.1038/nrcardio.2016.163 27708278 PMC5286912

[47] Pavo N, Prausmuller S, Spinka G, Goliasch G, Bartko PE, Wurm R, et al Myocardial angiotensin metabolism in end-stage heart failure. J Am Coll Cardiol 2021;77:1731–43. 10.1016/j.jacc.2021.01.052 33832600

[48] Jia G, Aroor AR, Hill MA, Sowers JR. Role of renin-angiotensin-aldosterone system activation in promoting cardiovascular fibrosis and stiffness. Hypertension 2018;72:537–48. 10.1161/HYPERTENSIONAHA.118.11065 29987104 PMC6202147

[49] Liu M, Li XC, Lu L, Cao Y, Sun RR, Chen S, et al Cardiovascular disease and its relationship with chronic kidney disease. Eur Rev Med Pharmacol Sci 2014;18:2918–26. 25339487

[50] de Denus S, O'Meara E, Desai AS, Claggett B, Lewis EF, Leclair G, et al Spironolactone metabolites in TOPCAT—new insights into regional variation. N Engl J Med 2017;376:1690–2. 10.1056/NEJMc1612601 28445660 PMC5590224

[51] Jhund PS, Talebi A, Henderson AD, Claggett BL, Vaduganathan M, Desai AS, et al Mineralocorticoid receptor antagonists in heart failure: an individual patient level meta-analysis. Lancet 2024;404:1119–31. 10.1016/s0140-6736(24)01733-1 39232490

[52] Hunter RW, Bailey MA. Hyperkalemia: pathophysiology, risk factors and consequences. Nephrol Dial Transplant 2019;34:iii2–iii11. 10.1093/ndt/gfz206 31800080 PMC6892421

[53] Desai AS, Liu J, Pfeffer MA, Claggett B, Fleg J, Lewis EF, et al Incident hyperkalemia, hypokalemia, and clinical outcomes during spironolactone treatment of heart failure with preserved ejection fraction: analysis of the TOPCAT trial. J Card Fail 2018;24:313–20. 10.1016/j.cardfail.2018.03.002 29572190

[54] U.S. Food and Drug Administration . *CAROSPIR (Spironolactone) Oral Suspension US PI*. 2021. https://www.accessdata.fda.gov/drugsatfda_docs/label/2021/209478Orig1s002lbl.pdf (24 February 2026, date last accessed).

[55] U.S. Food and Drug Administration . *INSPRA (Eplerenone) Tablets for Oral Use US PI*. 2018. https://www.accessdata.fda.gov/drugsatfda_docs/label/2018/021437s015lbl.pdf (24 February 2026, date last accessed).

[56] Gregg LP, Navaneethan SD. Steroidal or non-steroidal MRAs: should we still enable RAASi use through K binders? Nephrol Dial Transplant 2023;38:1355–65. 10.1093/ndt/gfac284 36264349 PMC10229268

[57] Arnold SV, Silverman DN, Gosch K, Nassif ME, Infeld M, Litwin S, et al Beta-blocker use and heart failure outcomes in mildly reduced and preserved ejection fraction. JACC Heart Fail 2023;11:893–900. 10.1016/j.jchf.2023.03.017 37140513

[58] Karakasis P, Pamporis K, Stachteas P, Patoulias D, Bougioukas KI, Fragakis N. Efficacy and safety of sodium-glucose cotransporter-2 inhibitors in heart failure with mildly reduced or preserved ejection fraction: an overview of 36 systematic reviews. Heart Fail Rev 2023;28:1033–51. 10.1007/s10741-023-10324-3 37284930

[59] Anker SD, Butler J, Usman MS, Filippatos G, Ferreira JP, Bocchi E, et al Efficacy of empagliflozin in heart failure with preserved vs mid-range ejection fraction: a pre-specified analysis of EMPEROR-preserved. Nat Med 2022;28:2512–20. 10.1038/s41591-022-02041-5 36471037 PMC9800272

[60] Butler J, Khan MS, Mori C, Filippatos GS, Ponikowski P, Comin-Colet J, et al Minimal clinically important difference in quality of life scores for patients with heart failure and reduced ejection fraction. Eur J Heart Fail 2020;22:999–1005. 10.1002/ejhf.1810 32239794

[61] Zinman B, Wanner C, Lachin JM, Fitchett D, Bluhmki E, Hantel S, et al Empagliflozin, cardiovascular outcomes, and mortality in type 2 diabetes. N Engl J Med 2015;373:2117–28. 10.1056/NEJMoa1504720 26378978

[62] Herrington WG, Staplin N, Wanner C, Green JB, Hauske SJ, Emberson JR, et al Empagliflozin in patients with chronic kidney disease. N Engl J Med 2023;388:117–27. 10.1056/NEJMoa2204233 36331190 PMC7614055

[63] Wiviott SD, Raz I, Bonaca MP, Mosenzon O, Kato ET, Cahn A, et al Dapagliflozin and cardiovascular outcomes in type 2 diabetes. N Engl J Med 2019;380:347–57. 10.1056/NEJMoa1812389 30415602

[64] Heerspink HJL, Stefansson BV, Correa-Rotter R, Chertow GM, Greene T, Hou FF, et al Dapagliflozin in patients with chronic kidney disease. N Engl J Med 2020;383:1436–46. 10.1056/NEJMoa2024816 32970396

[65] Neal B, Perkovic V, Mahaffey KW, de Zeeuw D, Fulcher G, Erondu N, et al Canagliflozin and cardiovascular and renal events in type 2 diabetes. N Engl J Med 2017;377:644–57. 10.1056/NEJMoa1611925 28605608

[66] Perkovic V, Jardine MJ, Neal B, Bompoint S, Heerspink HJL, Charytan DM, et al Canagliflozin and renal outcomes in type 2 diabetes and nephropathy. N Engl J Med 2019;380:2295–306. 10.1056/NEJMoa1811744 30990260

[67] American Diabetes Association Professional Practice Committee . 11. Chronic kidney disease and risk management: standards of care in diabetes-2025. Diabetes Care 2025;48:S239–51. 10.2337/dc25-S011 39651975 PMC11635029

[68] American Diabetes Association Professional Practice Committee . 10. Cardiovascular disease and risk management: standards of care in diabetes-2025. Diabetes Care 2025;48:S207–38. 10.2337/dc25-S010 39651970 PMC11635050

[69] Kidney Disease: Improving Global Outcomes (KDIGO) CKD Work Group . KDIGO 2024 clinical practice guideline for the evaluation and management of chronic kidney disease. Kidney Int 2024;105:S117–314. 10.1016/j.kint.2023.10.018 38490803

[70] McHugh K, DeVore AD, Wu J, Matsouaka RA, Fonarow GC, Heidenreich PA, et al Heart failure with preserved ejection fraction and diabetes: JACC state-of-the-art review. J Am Coll Cardiol 2019;73:602–11. 10.1016/j.jacc.2018.11.033 30732715

[71] Savarese G, Settergren C, Schrage B, Thorvaldsen T, Löfman I, Sartipy U, et al Comorbidities and cause-specific outcomes in heart failure across the ejection fraction spectrum: a blueprint for clinical trial design. Int J Cardiol 2020;313:76–82. 10.1016/j.ijcard.2020.04.068 32360702

[72] Patel RN, Sharma A, Prasad A, Bansal S. Heart failure with preserved ejection fraction with CKD: a narrative review of a multispecialty disorder. Kidney Med 2023;5:100705. 10.1016/j.xkme.2023.100705 38046909 PMC10692714

[73] Savarese G, Lindberg F, Filippatos G, Butler J, Anker SD. Mineralocorticoid receptor overactivation: targeting systemic impact with non-steroidal mineralocorticoid receptor antagonists. Diabetologia 2024;67:246–62. 10.1007/s00125-023-06031-1 38127122 PMC10789668

[74] Di Lullo L, Lavalle C, Scatena A, Mariani MV, Ronco C, Bellasi A. Finerenone: questions and answers-the four fundamental arguments on the new-born promising non-steroidal mineralocorticoid receptor antagonist. J Clin Med 2023;12:3992. 10.3390/jcm12123992 37373685 PMC10299719

[75] U.S. Food and Drug Administration . *KERENDIA (finerenone) tablets, for oral use*. 2025. https://www.accessdata.fda.gov/drugsatfda_docs/label/2025/215341s009lbl.pdf (30 May 2024, date last accessed).

[76] Bakris GL, Agarwal R, Anker SD, Pitt B, Ruilope LM, Rossing P, et al Effect of finerenone on chronic kidney disease outcomes in type 2 diabetes. N Engl J Med 2020;383:2219–29. 10.1056/NEJMoa2025845 33264825

[77] Pitt B, Filippatos G, Agarwal R, Anker SD, Bakris GL, Rossing P, et al Cardiovascular events with finerenone in kidney disease and type 2 diabetes. N Engl J Med 2021;385:2252–63. 10.1056/NEJMoa2110956 34449181

[78] Vaduganathan M, Claggett BL, Desai AS, Jhund PS, Lam CSP, Senni M, et al Estimated long-term benefits of finerenone in heart failure: a prespecified secondary analysis of the FINEARTS-HF randomized clinical trial. JAMA Cardiol 2025;10:176–81. 10.1001/jamacardio.2024.3782 39332395 PMC11581494

[79] Vaduganathan M, Claggett BL, Chatur S, Desai AS, Jhund PS, Vardeny O, et al Lifetime benefits of comprehensive medical therapy in heart failure with mildly reduced or preserved ejection fraction. Nat Med 2025;32:325–31. 10.1038/s41591-025-04037-3 41052644 PMC12823412

[80] ClinicalTrials.gov . *A Study to Evaluate Finerenone on Clinical Efficacy and Safety in Patients With Heart Failure Who are Intolerant or Not Eligible for Treatment With Steroidal Mineralocorticoid Receptor Antagonists (FINALITY-HF)*. 2024. https://classic.clinicaltrials.gov/ct2/show/NCT06033950 (24 February 2026, date last accessed).

[81] von Haehling S, Doehner W, Evertz R, Garfias-Veitl T, Derad C, Diek M, et al Ferric carboxymaltose and exercise capacity in heart failure with preserved ejection fraction and iron deficiency: the FAIR-HFpEF trial. Eur Heart J 2024;45:3789–800. 10.1093/eurheartj/ehae479 39185895 PMC11452748

[82] Anker SD, Usman MS, Anker MS, Butler J, Böhm M, Abraham WT, et al Patient phenotype profiling in heart failure with preserved ejection fraction to guide therapeutic decision making. A scientific statement of the Heart Failure Association, the European Heart Rhythm Association of the European Society of Cardiology, and the European Society of Hypertension. Eur J Heart Fail 2023;25:936–55. 10.1002/ejhf.2894 37461163

[83] Savarese G, Gatti P, Benson L, Adamo M, Chioncel O, Crespo-Leiro MG, et al Left ventricular ejection fraction digit bias and reclassification of heart failure with mildly reduced vs reduced ejection fraction based on the 2021 definition and classification of heart failure. Am Heart J 2024;267:52–61. 10.1016/j.ahj.2023.11.008 37972677

[84] Moriyama T, Schneider M, Virgitti JBJ, Peach E, Barone S, Kumar S, et al Undiagnosed stage 3 chronic kidney disease in patients with a history of heart failure: a report from REVEAL-CKD. J Am Coll Cardiol 2022;79:440. 10.1016/S0735-1097(22)01431-0

[85] Wittbrodt E, Kushner P, Barone S, Kumar S, Chen H, Jarbrink K, et al Prevalence of and factors associated with undiagnosed stage 3 chronic kidney disease in patient with a history of heart failure: a report from REVEAL-CKD. Eur Heart J 2021;42:ehab724.0831. 10.1093/eurheartj/ehab724.0831

[86] Mitsas AC, Elzawawi M, Mavrogeni S, Boekels M, Khan A, Eldawy M, et al Heart failure and cardiorenal syndrome: a narrative review on pathophysiology, diagnostic and therapeutic regimens—from a cardiologist’s view. J Clin Med 2022;11:7041. 10.3390/jcm11237041 36498617 PMC9741317

[87] Méndez AB, Azancot MA, Olivella A, Soler MJ. New aspects in cardiorenal syndrome and HFpEF. Clin Kidney J 2022;15:1807–15. 10.1093/ckj/sfac133 36158149 PMC9494528

[88] Tromp J, Shen L, Jhund PS, Anand IS, Carson PE, Desai AS, et al Age-related characteristics and outcomes of patients with heart failure with preserved ejection fraction. J Am Coll Cardiol 2019;74:601–12. 10.1016/j.jacc.2019.05.052 31370950

[89] Anderson L, Bayes-Genis A, Bodegard J, Mullin K, Gustafsson S, Rosano GMC, et al Suspected de novo heart failure in outpatient care: the REVOLUTION HF study. Eur Heart J 2025;46:1493–503. 10.1093/eurheartj/ehaf034 39935142 PMC12011520

[90] Saw EL, Ramachandran S, Valero-Muñoz M, Sam F. Skeletal muscle (dys)function in heart failure with preserved ejection fraction. Curr Opin Cardiol 2021;36:219–26. 10.1097/hco.0000000000000824 33394707 PMC7895420

[91] Yuasa N, Obokata M, Harada T, Kagami K, Sorimachi H, Saito Y, et al Characterization and prognostic importance of chronotropic incompetence in heart failure with preserved ejection fraction. J Cardiol 2024;83:113–20. 10.1016/j.jjcc.2023.06.014 37419310

[92] Tokarczyk W, Urban S, Patrzalek P, Stolarski L, Iwanek G, Szymanski O, et al Potential effects of beta-blockers in HFpEF. Heart Fail Rev 2025;30:357–64. 10.1007/s10741-024-10468-w 39625687 PMC11802620

[93] Palau P, Seller J, Dominguez E, Sastre C, Ramon JM, de L, et al Effect of beta-blocker withdrawal on functional capacity in heart failure and preserved ejection fraction. J Am Coll Cardiol 2021;78:2042–56. 10.1016/j.jacc.2021.08.073 34794685

[94] Judge PK, Tuttle KR, Staplin N, Hauske SJ, Zhu D, Sardell R, et al The potential for improving cardio-renal outcomes in chronic kidney disease with the aldosterone synthase inhibitor vicadrostat (BI 690517): a rationale for the EASi-KIDNEY trial. Nephrol Dial Transplant 2024;40:1175–86. 10.1093/ndt/gfae263 PMC12209857 39533115

[95] Abdelnabi M, Saleh Y, Almaghraby A, Girgis H, Gerges F. Sacubitril/valsartan: a new dawn has begun! a revisited review. Curr Cardiol Rev 2022;18:e310821195982. 10.2174/1573403(17666210831142452 34488614 PMC9615216

[96] Pugliese NR, Masi S, Taddei S. The renin-angiotensin-aldosterone system: a crossroad from arterial hypertension to heart failure. Heart Fail Rev 2020;25:31–42. 10.1007/s10741-019-09855-5 31512149

[97] Parichatikanond W, Duangrat R, Kurose H, Mangmool S. Regulation of β-adrenergic receptors in the heart: a review on emerging therapeutic strategies for heart failure. Cells 2024;13:1674. 10.3390/cells13201674 39451192 PMC11506672

[98] Dhalla NS, Bhullar SK, Adameova A, Mota KO, de Vasconcelos CML. Status of β(1)-adrenoceptor signal transduction system in cardiac hypertrophy and heart failure. Rev Cardiovasc Med 2023;24:264. 10.31083/j.rcm2409264 39076390 PMC11270071

[99] Gallo G, Volpe M. Potential mechanisms of the protective effects of the cardiometabolic drugs type-2 sodium-glucose transporter inhibitors and glucagon-like peptide-1 receptor agonists in heart failure. Int J Mol Sci 2024;25:2484. 10.3390/ijms25052484 38473732 PMC10931718

[100] Taktaz F, Fontanella RA, Scisciola L, Pesapane A, Basilicata MG, Ghosh P, et al Bridging the gap between GLP1-receptor agonists and cardiovascular outcomes: evidence for the role of tirzepatide. Cardiovasc Diabetol 2024;23:242. 10.1186/s12933-024-02319-7 38987789 PMC11238498

[101] U.S. Food and Drug Administration . *Wegovy (semaglutide) US PI*. 2024. https://www.accessdata.fda.gov/drugsatfda_docs/label/2024/215256s011lbl.pdf (24 February 2026, date last accessed).

[102] Lam CSP, Køber L, Kuwahara K, Lund LH, Mark PB, Mellbin LG, et al Balcinrenone plus dapagliflozin in patients with heart failure and chronic kidney disease: results from the phase 2b MIRACLE trial. Eur J Heart Fail 2024;26:1727–35. 10.1002/ejhf.3294 38783712

[103] ClinicalTrials.gov . *Study to Evaluate the Effect of Balcinrenone/Dapagliflozin in Patients With Heart Failure and Impaired Kidney Function (BalanceD-HF)*. 2024. https://clinicaltrials.gov/study/NCT06307652 (24 February 2026, date last accessed).

[104] ClinicalTrials.gov . *A Study to Test Whether Vicadrostat in Combination With Empagliflozin Helps People With Heart Failure*. 2024. https://clinicaltrials.gov/study/NCT06424288 (24 February 2026, date last accessed).

[105] Kosiborod MN, Abildstrom SZ, Borlaug BA, Butler J, Rasmussen S, Davies M, et al Semaglutide in patients with heart failure with preserved ejection fraction and obesity. N Engl J Med 2023;389:1069–84. 10.1056/NEJMoa2306963 37622681

[106] Kosiborod MN, Petrie MC, Borlaug BA, Butler J, Davies MJ, Hovingh GK, et al Semaglutide in patients with obesity-related heart failure and type 2 diabetes. N Engl J Med 2024;390:1394–407. 10.1056/NEJMoa2313917 38587233

[107] Butler J, Shah SJ, Petrie MC, Borlaug BA, Abildstrøm SZ, Davies MJ, et al Semaglutide versus placebo in people with obesity-related heart failure with preserved ejection fraction: a pooled analysis of the STEP-HFpEF and STEP-HFpEF DM randomised trials. Lancet 2024;403:1635–48. 10.1016/s0140-6736(24)00469-0 38599221 PMC11317105

[108] Petrie MC, Borlaug BA, Butler J, Davies MJ, Kitzman DW, Shah SJ, et al Semaglutide and NT-proBNP in obesity-related HFpEF: insights from the STEP-HFpEF program. J Am Coll Cardiol 2024;84:27–40. 10.1016/j.jacc.2024.04.022 38819334

[109] Packer M, Zile MR, Kramer CM, Baum SJ, Litwin SE, Menon V, et al Tirzepatide for heart failure with preserved ejection fraction and obesity. N Engl J Med 2024;392:427–37. 10.1056/NEJMoa2410027 39555826

[110] Bamberg K, Johansson U, Edman K, William-Olsson L, Myhre S, Gunnarsson A, et al Preclinical pharmacology of AZD9977: a novel mineralocorticoid receptor modulator separating organ protection from effects on electrolyte excretion. PLoS One 2018;13:e0193380. 10.1371/journal.pone.0193380 29474466 PMC5825103

[111] Pitt B, Williams GH. Aldosterone synthase inhibitors and mineralocorticoid receptor antagonists: competitors or collaborators? Circulation 2024;149:414–6. 10.1161/CIRCULATIONAHA.123.066314 38315762

[112] Ryan DH, Lingvay I, Deanfield J, Kahn SE, Barros E, Burguera B, et al Long-term weight loss effects of semaglutide in obesity without diabetes in the SELECT trial. Nat Med 2024;30:2049–57. 10.1038/s41591-024-02996-7 38740993 PMC11271387

[113] Wilding JPH, Batterham RL, Calanna S, Davies M, Van Gaal LF, Lingvay I, et al Once-weekly semaglutide in adults with overweight or obesity. N Engl J Med 2021;384:989–1002. 10.1056/NEJMoa2032183 33567185

[114] Marx N, Husain M, Lehrke M, Verma S, Sattar N. GLP-1 receptor agonists for the reduction of atherosclerotic cardiovascular risk in patients with type 2 diabetes. Circulation 2022;146:1882–94. 10.1161/circulationaha.122.059595 36508493

[115] Ferreira JP, Saraiva F, Sharma A, Vasques-Novoa F, Angelico-Goncalves A, Leite AR, et al Glucagon-like peptide 1 receptor agonists in patients with type 2 diabetes with and without chronic heart failure: a meta-analysis of randomized placebo-controlled outcome trials. Diabetes Obes Metab 2023;25:1495–502. 10.1111/dom.14997 36722252

[116] Shah SJ, Sharma K, Borlaug BA, Butler J, Davies M, Kitzman DW, et al Semaglutide and diuretic use in obesity-related heart failure with preserved ejection fraction: a pooled analysis of the STEP-HFpEF and STEP-HFpEF-DM trials. Eur Heart J 2024;45:3254–69. 10.1093/eurheartj/ehae322 38739118 PMC11400859

[117] Kramer CM, Borlaug BA, Zile MR, Ruff D, DiMaria JM, Menon V, et al Tirzepatide reduces LV mass and paracardiac adipose tissue in obesity-related heart failure. J Am Coll Cardiol 2025;85:699–706. 10.1016/j.jacc.2024.11.001 39566869

[118] Shahid I, Khan MS, Butler J, Fonarow GC, Greene SJ. Initiation and sequencing of guideline-directed medical therapy for heart failure across the ejection fraction spectrum. Heart Fail Rev 2025;30:515–23. 10.1007/s10741-025-10481-7 39815071

[119] Greene SJ, Butler J, Fonarow GC. Simultaneous or rapid initiation of combination therapy for heart failure with preserved ejection fraction. JAMA Cardiol 2025;10:407–8. 10.1001/jamacardio.2025.0038 40042835

[120] Rosano GMC, Vitale C, Spoletini I. Precision cardiology: phenotype-targeted therapies for HFmrEF and HFpEF. Int J Heart Fail 2024;6:47–55. 10.36628/ijhf.2023.0058 38694928 PMC11058434

[121] Desai N, Olewinska E, Famulska A, Remuzat C, Francois C, Folkerts K. Heart failure with mildly reduced and preserved ejection fraction: a review of disease burden and remaining unmet medical needs within a new treatment landscape. Heart Fail Rev 2024;29:631–62. 10.1007/s10741-024-10385-y 38411769 PMC11035416

[122] Rasalam R, Sindone A, Deed G, Audehm RG, Atherton JJ. State of precision medicine for heart failure with preserved ejection fraction in a new therapeutic age. ESC Heart Fail 2025;12:1544–57. 10.1002/ehf2.15205 39844745 PMC12055434

